# Melatonin suppresses serum starvation-induced autophagy of ovarian granulosa cells in premature ovarian insufficiency

**DOI:** 10.1186/s12905-022-02056-7

**Published:** 2022-11-24

**Authors:** Di Wu, Wenjie Zhao, Chengjuan Xu, Xin Zhou, Xia Leng, Yanmin Li

**Affiliations:** 1 Department of Reproductive Medicine, Weifang People’s Hospital, No.151 Guangwen Street, Kuiwen DistrictShandong Province, Weifang City, 261041 China; 2 Department of Gynecology, Shouguang People’s Hospital, Weifang, 262700 Shandong China; 3 Quality Management Office of Weifang People’s Hospital, Weifang, 262700 China

**Keywords:** Premature ovarian insufficiency, Granulosa cells, Melatonin, miR-15a-5p, Stat3, PI3K-Akt-mTOR pathway, Autophagy, Serum starvation

## Abstract

**Objectives:**

Premature ovarian insufficiency (POI) refers to the decline and cessation of ovarian functions in women under 40 years of age. Melatonin (MT) acts as a protective for the ovary. This study elucidated the role of MT in autophagy of granulosa cells (GCs) in POI via modulating the phosphatidylinositol-3-kinase (PI3K)-Akt-mammalian target of rapamycin (mTOR) pathway.

**Methods:**

The expression levels of microRNA (miR)-15a-5p, signal transducer and activator of transcription 3 (Stat3), and relevant hormones in the clinically collected serum samples of POI patients and healthy controls were examined. Human ovarian granulosa-like tumor cells (KGN) underwent serum starvation (SS) treatment to induce POI cell models and then received MT treatment. The expression levels of miR-15a-5p, Stat3, p-PI3K/PI3K, p-Akt/Akt, and p-mTOR/mTOR in KGN cells were tested via quantitative real-time polymerase chain reaction and Western blotting. KGN cell viability was assessed by MTT assay and the protein levels of autophagy-related markers Beclin-1, microtubule-associated protein light chain 3 II/I, and p62 were detected by Western blotting. The binding relation between miR-15a-5p and Stat3 was verified via the dual-luciferase reporter gene assay. Functional rescue experiments were performed to probe the underlying role of miR-15a-5p/Stat3/the PI3K-Akt-mTOR pathway in KGN cell autophagy.

**Results:**

miR-15a-5p was increased whilst Stat3 was decreased in the serum of POI patients and SS-induced KGN cells. MT inhibited miR-15a-5p and Stat3, activated the PI3K-Akt-mTOR pathway, and repressed cell autophagy in SS-induced KGN cells. miR-15a-5p targeted and repressed Stat3 expression. Upregulation of miR-15a-5p or downregulation of Stat3 or the PI3K-Akt-mTOR pathway promoted KGN cell autophagy.

**Conclusion:**

MT suppressed miR-15a-5p and activated Stat3 and the PI3K-Akt-mTOR pathway, finally impeding SS-induced autophagy of GCs.

**Supplementary Information:**

The online version contains supplementary materi1al available at 10.1186/s12905-022-02056-7.

## Introduction

Premature ovarian insufficiency (POI) refers to a heterogeneous disorder occurring in the ovary, biochemically featured by the depletion of follicles and the deficiency of ovarian estrogen and ovarian reserve, resulting in ovarian dysfunction and the onset of menopause [1]. By and large, about 1% women suffer from POI before their forties and 0.1% before their thirties [2]. Granulosa cells (GCs) are essential to maintain ovarian functions, including folliculogenesis, follicular atresia, and oocyte growth and quality [3, 4]. Existing studies have demonstrated that the dysfunction of GCs leads to disorders concerning ovarian function, such as POI and polycystic ovary syndrome (PCOS) [5, 6]. Autophagy is defined as a complex and evolutionarily conserved mechanism of intracellular recycle or degradation of eukaryotic components [7]. Specifically, autophagosomes fuse with lysosomes to form autophagolysosome to further degrade the components of the inclusions, maintaining cellular homeostasis and organelle renewal [8, 9]. Meanwhile, GC autophagy has been demonstrated to be suppressed by gonadotropins and further impart a profound effect on folliculogenesis and reproductive outcomes [10]. Therefore, we determined to explore the molecular mechanism of GC autophagy in POI.

Melatonin (MT) is a pleiotropic indolamine mainly synthesized by the pineal gland, bone marrow, retina, lymphocytes, and gastrointestinal tract [11]. Significantly, MT acts an anti-oxidant to protect oocytes and granulosa cells against oxidative damage, and has the potential to prevent ovarian aging [12]. Moreover, MT combined with estrogen-progestogen is conducive to improving infertility in POI patients [13], and MT limits oxidative stress-triggered GC autophagy via suppressing c-Jun N-terminal kinase [14]. Nonetheless, knowledges about the mechanism of MT in GC autophagy in POI are limited.

MicroRNAs (miRs) are well-studied endogenous noncoding RNAs with approximately 21 or 22 nucleotides (nt) in length and their emergence is recognized as a pivotal mechanism that modulates the post‐transcriptional regulation of gene expression [15]. miRs are known to affect GC apoptosis, oocyte maturation and apoptosis, and primordial follicle recruitment in POI patients [16]. Furthermore, miR-15a-5p expression is evident of adverse ovarian responses in human follicular fluid [17]. However, it is unknown whether miR-15a-5p has an impact on GC autophagy in POI.

Signal transducer and activator of transcription (Stat), composed of 7 members, is a group of transcription factors that participate in various cellular processes, such as normal cell proliferation, differentiation, and angiogenesis [18]. Emerging studies have reported the involvement of Stat3 in the pathogenesis of POI induced by 4-vinylcyclohexene diepoxide and its regulatory role in ovarian carcinoma cell autophagy [19] [20]. On another note, the phosphatidylinositol-3-kinase (PI3K)- serine/threonine kinase (Akt)-mammalian target of rapamycin (mTOR) pathway can be initiated by multiple molecules and play an indispensable role in maintaining cellular homeostasis through regulating cell cycle, survival, metabolism, and in particular, autophagy [21, 22]. Notably, MT can regulate the PI3K-Akt-mTOR pathway to affect autophagy in spinal cord injury [23], and the pathway mediated by miR-18b-5p/PTEN can mitigate the progression of PCOS [24]. Therefore, the aim of this work was to expound whether MT can regulate GC autophagy in POI via modulating miR-15a-5p/Stat3 and the PI3K-Akt-mTOR pathway, and to develop novel treatment pathways for POI.

## Materials and methods

### Study population

From March 2019 to March 2021, 32 POI patients (aged 18 to 40 years) who were admitted to Weifang People's Hospital were included. According to the healthy physical examination results, a total of 40 participants (aged 22 to 42 years) of reproductive age with normal menstrual cycle were included as the Control group. The study protocols were in accordance with the Declaration of Helsinki and were approved by the Ethics Committee of Weifang People's Hospital. Each participants signed an informed consent form.

### Inclusion criteria

The diagnosis of POI was conducted according to the diagnostic criteria of the European Society of Human Reproduction and Embryology [25]. The inclusion criteria employed in this work were as follows: age under 40 years at first diagnosis; oligomenorrhea or amenorrhea for at least 4 months; the increased levels of follicle-stimulating hormone (FSH; > 25 IU/L) in two measurements taken more than 4 weeks apart. Participants in the Control group were included based on the results of the routine physical examinations, and matched to the POI group according to body mass index (BMI) and age (± 3 years).

The employed exclusion criteria were as follows: autoimmune diseases; chronic diseases (including renal diseases, hepatic diseases, hypertension, congestive heart failure, and cerebrovascular or cardiovascular diseases); history of treatments including oophorectomy, hysterectomy, radiotherapy, chemotherapy, or hormonal replacement therapy. As well, participants who were matched the following criteria were excluded: smoking; taking vitamin supplements; and currently during pregnancy or breastfeeding.

Every participant was asked to complete a questionnaire, and the responses were collected and analyzed as the available data in this study. Detailed information about the lifestyle factors, social demographic data, gynecological history, reproductive history, and other medical history of every participant was obtained in the form of face-to-face interviews with the participant by trained interviewers. BMI of every participant was calculated and recorded based on the age, height, and weight, and BMI in the range of 18.5 to 23.9 kg/m^2^ was determined as normal weight while BMI > 30.0 kg/m^2^ was determined as obesity.

### Blood collection and hormone measurement

All patients (fasting more than 8 h) received blood collection in the early morning.

With no consideration of the patient’s menstrual cycle, peripheral blood (PB) samples (PBSs) were collected from POI patients. Venous blood (VB) samples of participants in the Control group were collected at the early follicular phase of the menstrual cycle (day 1 to day 5 of spontaneous bleeding episode) to measure the basal levels of hormones FSH, luteinizing hormone (LH), estradiol (E2), and anti-Müllerian hormone (AMH). The collection and use of blood samples were performed strictly following the instructions and specifications provided by the manufacturers. VB samples were centrifuged at 4℃ for to separate blood serum and immediately preserved at -80℃.

Thereafter, the levels of FSH, LH, E2, and AMH in serum were examined by an automatic Roche Modular Analytics E170 IA system (Roche Diagnostics, Mannheim, Germany).

### Cell culture

The undifferentiated human ovarian granulosa-like tumor KGN cells obtained from Procell Life Science &Technology Co., Ltd. (Wuhan, Hubei, China) were inoculated in Dulbecco’s modified Eagle’s medium (DMEM)/F12 (Sigma, St. Louis, MO, USA) adding with 10% fetal bovine serum (FBS; Hyclone Laboratories, South Logan, UT, USA) and a combination of 100 U/mL penicillin and 0.1 mg/mL streptomycin (Invitrogen, Carlsbad, CA, USA) in a humidified incubator at 37℃ with 5% CO_2_.

### Serum starvation (SS) treatment

After 48 h of culture, KGN cells were rinsed twice with 5 mL phosphate-buffered saline (PBS) to completely clear the medium. Then, the culture plates were added with 5 mL low-serum culture medium (DMEM/F12 medium with 0.1% FBS). And KGN cells were incubated in an incubator for 12 h, after which KGN cells were treated with 100 pM melatonin (MT; Sigma) [26] or 0.5 μM LY294002 (MCE, Monmouth Junction, NJ, USA) 24 h before SS treatment.

### Cell transfection

miR-15a-5p-mimic, si-Stat3-1, si-Stat3-2, and their respective negative controls (mimic-NC and si-NC) were designed and synthesized by Genepharma (Shanghai, China), and based on the manufacturer’s requirements, these plasmids were transfected into KGN cells using Lipofectamine 3000 (Thermo Fisher Scientific, Waltham, MA, USA) 24 h before SS treatment.

### 3-(4,5-dimethyl-2-thiazolyl)-2, 5-diphenyl-2-H-tetrazolium bromide (MTT) assay

MTT assay was conducted to determine KGN cell viability. Briefly, KGN cells in different treatment groups were seeded at a density of 1 × 10^4^ cells/well in 96-well plates, and each well was added with 20 μL MTT solution (Sigma) at 24 h, 48 h, and 72 h for 4 h of incubation. Then, cells were added with 150 μL dimethyl sulfoxide (DMSO; Sigma) and shaken for 10 min. Last, the value of optical density (OD) at a wavelength of 490 nm were measured using a microplate reader.

### Quantitative real-time polymerase chain reaction (qRT-PCR)

Based on the manufacturer’s protocols, the total RNA of serum samples or KGN cells was separated using the TRIzol reagent (Invitrogen) and quantified using the OD ratio (OD260/OD280). The total RNA (1 μg) was reverse-transcribed into the complementary DNA using a QuantiTect Reverse-Transcription kit (Qiagen, Duesseldorf, Germany). Thereafter, qPCR was performed on ABI7500 R-T PCR System (Applied Biosystems, Foster City, CA, USA) using AceQ qPCR SYBR Green Master Mix (Vazyme, Nanjing, Jiangsu, China). Primers sequences in qPCR are presented in Table 1. The relative expression of genes was processed using 2^−△△Ct^ method and normalized to U6 [27] and GAPDH.

**Table 1:** qPCR primers

**Gene**	**Forward Primer (**5′-3′**)**	**Reverse Primer (**5′-3′**)**
*miR-15a-5p*	GCCGAGTAGCAGCACAUA	CTCAACTGGTGTCGTGGA
*Stat3*	AGAAACAGGATGGCCCAA	CTCAGCTCCTCACATGGG
U6	GTGCTCGCTTCGGCAGCA	AAAATATGGAACGCTTCA
GAPDH	CTCAACTACATGGTTTAC	CCAGGGGTCTTACTCCTT

### Western blotting

KGN cells were lysed using RIPA lysis buffer (Thermo Fisher Scientific) to isolate the total RNA, and the protein concentration was determined using the bicinchoninic acid protein determination assay. Thereafter, protein samples were isolated using 10% sodium dodecyl sulphate–polyacrylamide gel electrophoresis and then transferred onto polyvinylidene fluoride membranes, after which 5% skim milk was used to block the membranes. Subsequently, the membranes were incubated with the following antibodies: anti-Stat3 (1:1000; ab68153; Abcam, Cambridge, MA, USA), anti-Beclin-1 (1:2000; ab207612; Abcam), anti-microtubule-associated protein light chain 3 (LC3) II/I (1:2000; ab192890; Abcam), anti-p62 (1:10000; ab109012; Abcam), anti-p-PI3K (1:500; ab182651; Abcam), anti-phosphatidylinositol-3-kinase (PI3K; 1:1000; ab191606; Abcam), anti-p-Akt (1:500; ab38449; Abcam), anti-Akt (1:1000; ab179463; Abcam), anti-p-mTOR (1:1000; ab109268; Abcam), anti-mTOR (1:1000; ab32028; Abcam) and anti-GAPDH (1:2500; ab9485; Abcam) overnight at 4℃. After washed thrice, the membranes were incubated with horse radish peroxidase-conjugated anti-IgG at room temperature for 1 h. After rinsing thrice with PBS containing Tween-20 (PBST), the protein brands were developed using the enhanced-chemiluminescence reagent (Millipore, Billerica, MA, USA) and analyzed using ImageJ software (NIH, Bethesda, MD, USA). Because of the experimental fund, we performed cleavage before antibody incubation. The original images of the blots including all replicates were shown in the supplementary information.

### Dual-luciferase reporter gene assay

Stat3 3’UTR sequence containing miR-15a-5p binding sites was inserted into the pmirGLO vector to construct Stat3-wild type (Stat3-WT). Meanwhile, Stat3-mutant type (Stat3-MT) plasmid containing the target sites was also constructed. The above plasmids were transfected into KGN cells with miR-15a-5p or mimic-NC using Lipofectamine 3000 (Invitrogen). Based on the manufacturer’s protocols, the luciferase activity was examined using a dual-luciferase reporter gene assay kit (Solarbio, Beijing, China).

### Statistical analysis

Analysis and mapping for experimental data were conducted using GraphPad Prism 8.0 software (GraphPad Software Inc., San Diego, CA, USA). Student’s *t*-test was employed to compare the differences between 2 groups; One-way analysis of variance (ANOVA) and two-way ANOVA were adopted to compare the differences among multiple groups and Tukey’s post-hoc was conducted for post-test of data; Pearson correlation analysis was employed to analyze the correlation between serum miR-15a-5p expression and serum Stat3 expression; Measurement data were exhibited as mean ± standard deviation (SD); *p* < 0.05 indicated a statistical significance and *p* < 0.01 indicated a highly statistical significance.

## Results

### miR-15a-5p is highly expressed in POI patients

**Fig. 1:**
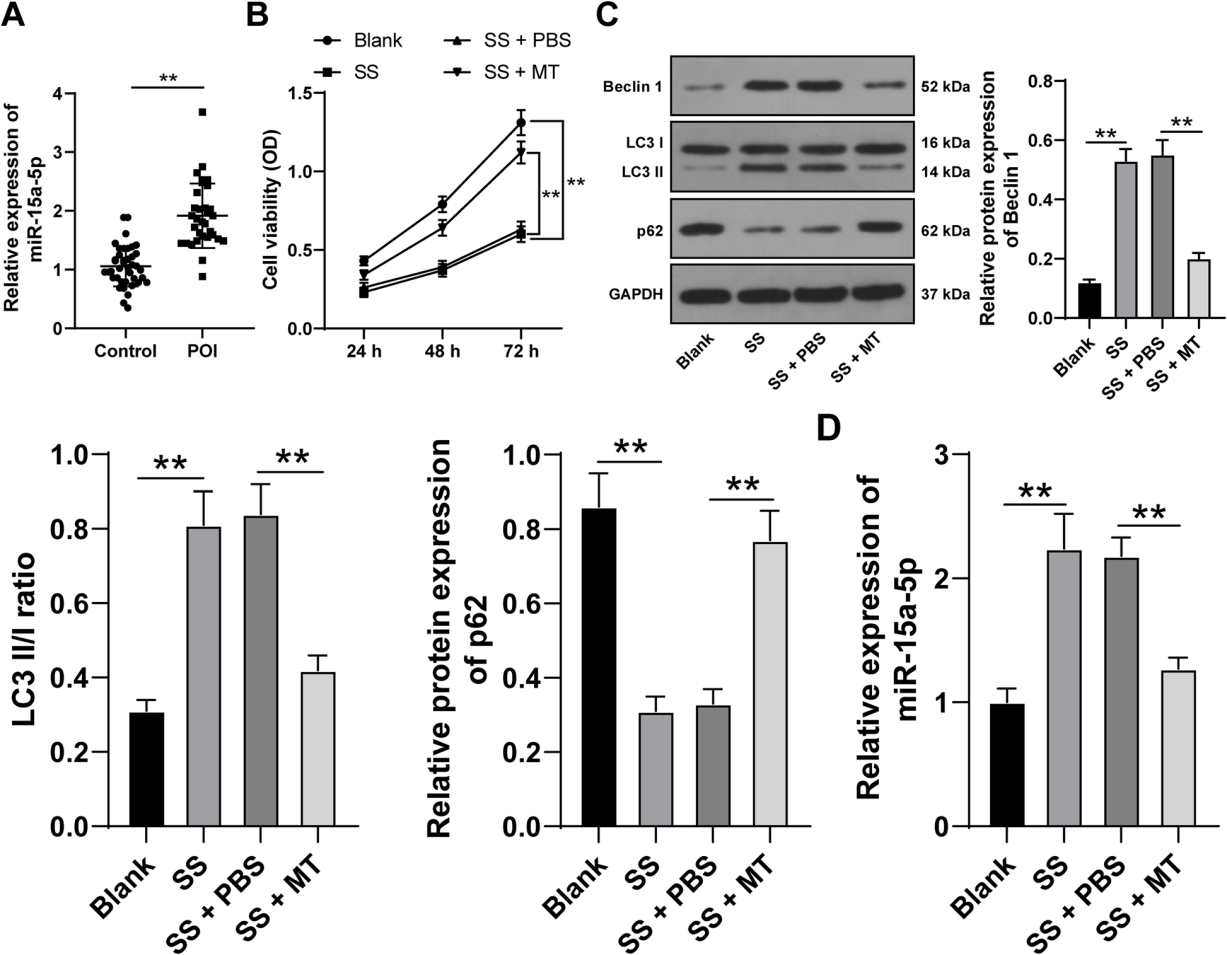
MT limits SS-induced KGN cell autophagy and downregulates miR-15a-5p. **A** The expression levels of miR-15a-5p in the serum of the healthy controls (*n* = 40) and POI patients (*n* = 32) were examined using qRT-PCR. KGN cells were treated with serum starvation (SS) to induce autophagy and then treated with melatonin (MT). **B** KGN cell viability was detected via MTT assay; **C** The expression levels of autophagy-related proteins Beclin 1, LC3 II/I, and p62 in KGN cells were examined via Western blotting; **D** The expression levels of miR-15a-5p in KGN cells were examined via qRT-PCR. Experiments were performed in 3 independent repetitions; experimental data were presented as mean ± SD; data in figure A were examined via the *t*-teat; data in figure B were examined via two-way ANOVA; data in figures C-D were examined via one-way ANOVA, followed by Tukey’s post-hoc test; ** *p* < 0.01

In this prospective study, 32 POI patients were enrolled as the POI group while 40 healthy participants as the Control group. The demographics characteristics and biochemical parameters of all participants are listed in Table 2. There were no significant differences in terms of age and BMI at enrollment between the POI group and the Control group. Compared with controls, the serum FSH and LH levels were higher in POI patients, while the serum levels of AMH were lower in POI patients. Besides, miR-15a-5p expression levels in POI patients were markedly increased compared to the controls (*p* < 0.05, Fig. 1A).

**Table 2:** Clinical characteristics and biochemical parameters of the participants

Variables	Control group (*n* = 40)	POI group (*n* = 32)	*p*-Value
Age, years	32.93 ± 5.55	31.13 ± 4.98	0.1570
BMI, kg/m^2^	20.84 ± 3.12	20.75 ± 2.82	0.8994
FSH, IU/L	7.29 ± 2.29	81.54 ± 31.85	< 0.0001
LH, IU/L	4.55 ± 1.43	44.04 ± 15.76	< 0.0001
E2, pmol/L	168.35 ± 73.89	201.24 ± 105.36	0.1246
AMH, ng/mL	3.87 ± 2.23	0.03 ± 0.02	< 0.0001

Data were analyzed using the *t*-test; data were expressed as mean ± standard deviation; *p* < 0.05 indicated statistical significance. *POI* premature ovarian insufficiency, *BMI* body mass index, *FSH* follicle-stimulating hormone, *LH* luteinizing hormone, *E2* estradiol, *AMH*, anti-Müllerian hormone

### MT limits SS-induced KGN cell autophagy and downregulates miR-15a-5p

Firstly, to investigate the effect of MT on autophagy of ovarian GCs in POI, KGN cell autophagy models were established through SS treatment. In KGN cells induced by SS, KGN cell viability was reduced (*p* < 0.05, Fig. 1B), and Beclin-1 protein levels and LC3 II/I ratio were increased while p62 protein levels was declined (*p* < 0.05, Fig. 1C). However, after MT treatment in SS-induced KGN cells, KGN cell viability was increased (*p* < 0.05, Fig. 1B), and Beclin-1 protein levels and LC3 II/I ratio were decreased while p62 protein levels were increased (*p* < 0.05, Fig. 1C). Preceding studies illustrated that miR-15a induces autophagy and participates in the progression of POI [28, 29]. Afterwards, qRT-PCR revealed upregulation of miR-15a-5p in SS-induced KGN cells and downregulation of miR-15a-5p in the cells after MT treatment (*p* < 0.05, Fig. 1D). Collectively, these results indicated that MT exerted effects on restraining SS-induced KGN cell autophagy and miR-15a-5p expression.

### miR-15a-5p upregulation boosts SS-induced KGN cell autophagy

**Fig. 2:**
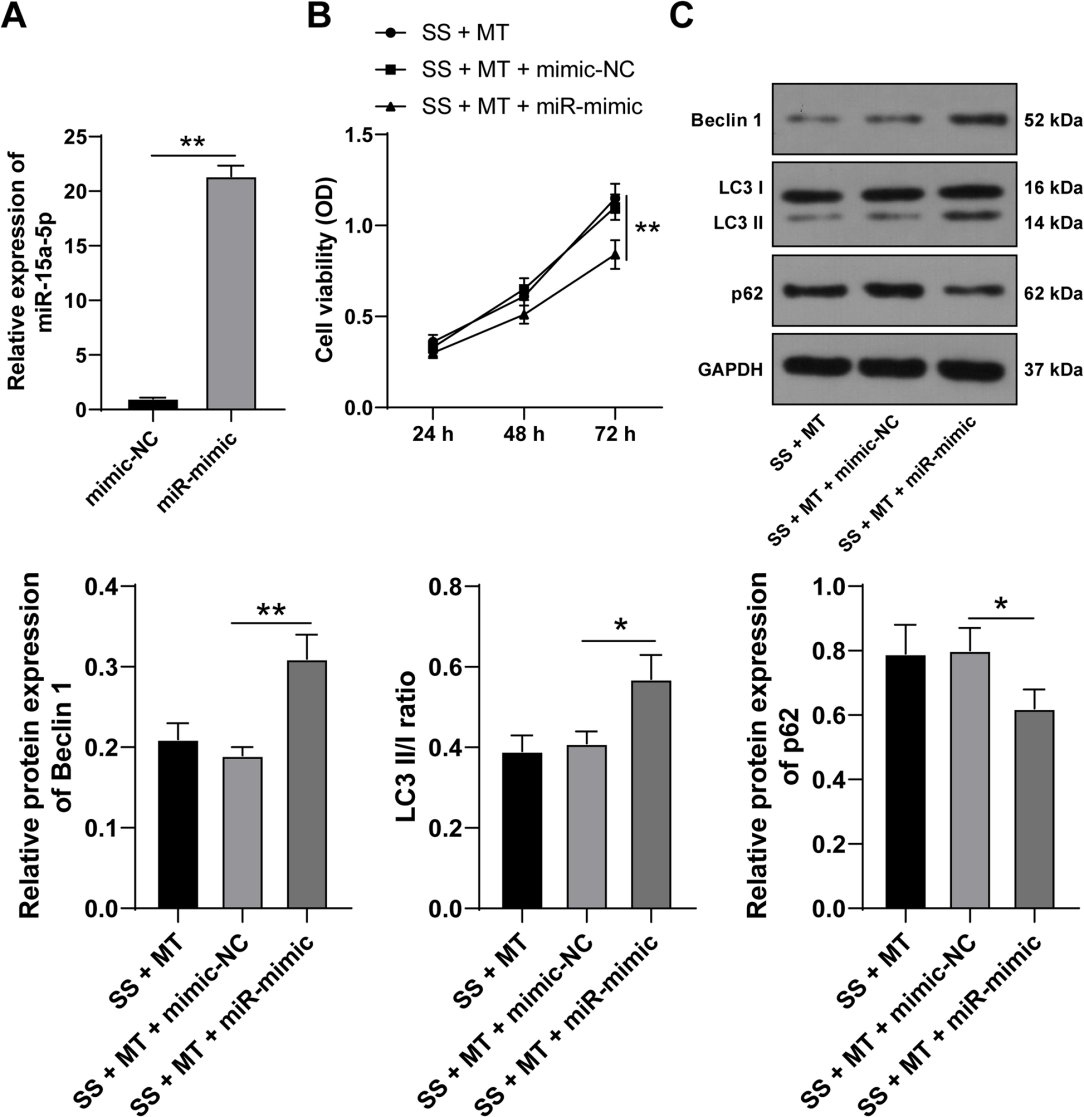
miR-15a-5p upregulation boosts SS-induced KGN cell autophagy. KGN cells were transfected with plasmid miR-15a-5p-mimic (miR-mimic), with mimic-NC as a control. **A** The transfection efficiency was examined via qRT-PCR. KGN cells with miR-15a-5p-mimic were combined with MT treatment. **B** KGN cell viability was detected via MTT assay; **C** The expression levels of autophagy-related proteins Beclin 1, LC3 II/I, and p62 in KGN cells were examined via Western blotting. Experiments were performed in 3 independent repetitions; experimental data were presented as mean ± SD; data in figure A were examined via Student’s *t*-test and data in figures B-C were examined via one-way ANOVA, followed by Tukey’s post-hoc test; * *p* < 0.05 and ** *p* < 0.01

To verify whether MT regulates GC autophagy with the help of miR-15a-5p, we upregulated miR-15a-5p expression in KGN cells using miR-15a-5p-mimic (*p* < 0.05, Fig. 2A), and KGN cells overexpressing miR-15a-5p were combined with MT treatment to perform the rescue experiment. Compared with the SS + MT + mimic-NC group, miR-15a-5p overexpression reduced KGN cell viability (*p* < 0.05, Fig. 2B), elevated Beclin-1 protein levels and LC3 II/I ratio, and decreased p62 protein levels (*p* < 0.05, Fig. 2C). Together, the above findings suggested that miR-15a-5p overexpression averted the repressive role of MT in SS-induced KGN cell autophagy.

### miR-15a-5p binds to Stat3

**Fig. 3:**
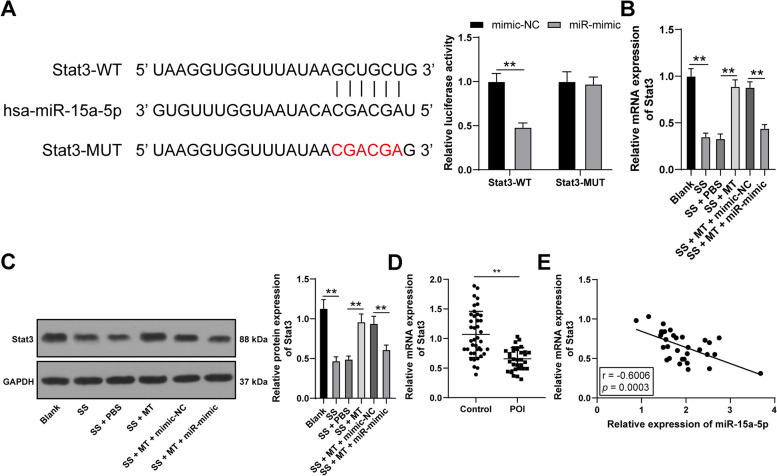
miR-15a-5p binds to Stat3. **A** The binding association between miR-15a-5p and Stat3 was verified via dual-luciferase reporter gene assay; **B**-**C** Stat3 expression levels in KGN cells were examined via qRT-PCR and Western blotting; **D** The expression levels of Stat3 in the serum of the healthy controls (*n* = 40) and POI patients (*n* = 32) were examined using qRT-PCR; **E** The correlation between serum miR-15a-5p expression and serum Stat3 expression was analyzed by Pearson correlation analysis. Experiments were performed in 3 independent repetitions; experimental data were presented as mean ± SD; data in figure A were examined via two-way ANOVA and data in figures B-C were examined via one-way ANOVA, followed by Tukey’s post-hoc test; ** *p* < 0.01

It has been observed that Stat3 is under-expressed in POI [30]. We predicted the potential binding sites between miR-15a-5p and Stat3 via Starbase and verify the binding association between miR-15a-5p and Stat3 via the dual-luciferase reporter gene assay (*p* < 0.05, Fig. 3A). Additionally, Stat3 expression levels in KGN cells were examined and it was found that Stat3 was downregulated in SS-induced KGN cells. However, Stat3 expression levels in the cells were up-regulated after MT treatment and decreased after miR-15a-5p overexpression (*p* < 0.05, Fig. 3B-C). Besides, the mRNA levels of Stat3 were declined in serum of POI patients (*p* < 0.05, Fig. 3D), and were negatively correlated to miR-15a-5p expression levels (*p* < 0.05, Fig. 3E). These data suggested that miR-15a-5p targeted and repressed Stat3 expression.

### Silencing Stat3 neutralizes the repression of SS-induced KGN cell autophagy caused by MT

**Fig. 4:**
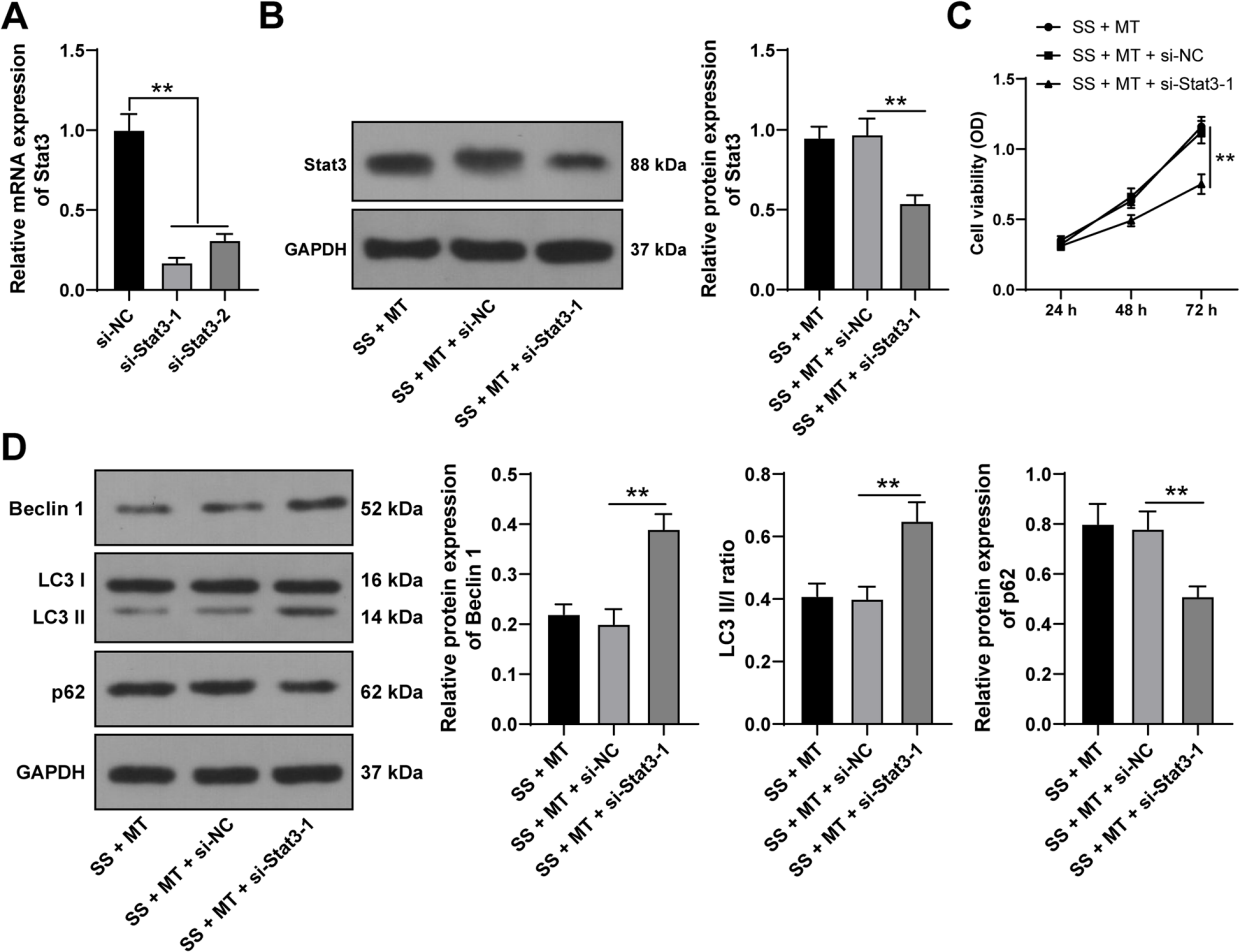
Silencing Stat3 neutralizes the repression of MT on SS-induced KGN cell autophagy. KGN cells were transfected with si-Stat3-1 and si-Stat3-2, with si-NC as a negative control. **A** The transfection efficiency was examined via qRT-PCR. si-Stat3-1 with better silencing effect was chosen for combined treatment with MT. **B** The protein levels of Stat3 in KGN cells were determined via Western blotting; **C** KGN cell viability was detected via MTT assay; **D** The expression levels of autophagy-related proteins Beclin 1, LC3 II/I, and p62 in KGN cells were examined via Western blotting. Experiments were performed in 3 independent repetitions; experimental data were presented as mean ± SD; data in figure C were examined via two-way ANOVA and data in figures **A**-**B** and **D** were examined via one-way ANOVA, followed by Tukey’s post-hoc test; ** *p* < 0.01

Thereafter, we suppressed Stat3 expression in KGN cells using si-Stat3-1 and si-Stat3-2 (*p* < 0.05, Fig. 4A). Then, si-Stat3-1 was found to have higher silencing efficiency, and as a result was selected for the rescue experiment with MT treatment. After Stat3 knockdown, Stat3 protein levels in KGN cells were decreased (*p* < 0.05, Fig. 4B), KGN cell viability was reduced (*p* < 0.05, Fig. 4C), Beclin-1 protein levels and LC3 II/I ratio were increased, and p62 protein levels were declined (*p* < 0.05, Fig. 4D). Overall, Stat3 knockdown reversed the repressive role of MT in SS-induced KGN cell autophagy.

### MT activates the PI3K-Akt-mTOR pathway via miR-15a-5p/Stat3

**Fig. 5:**
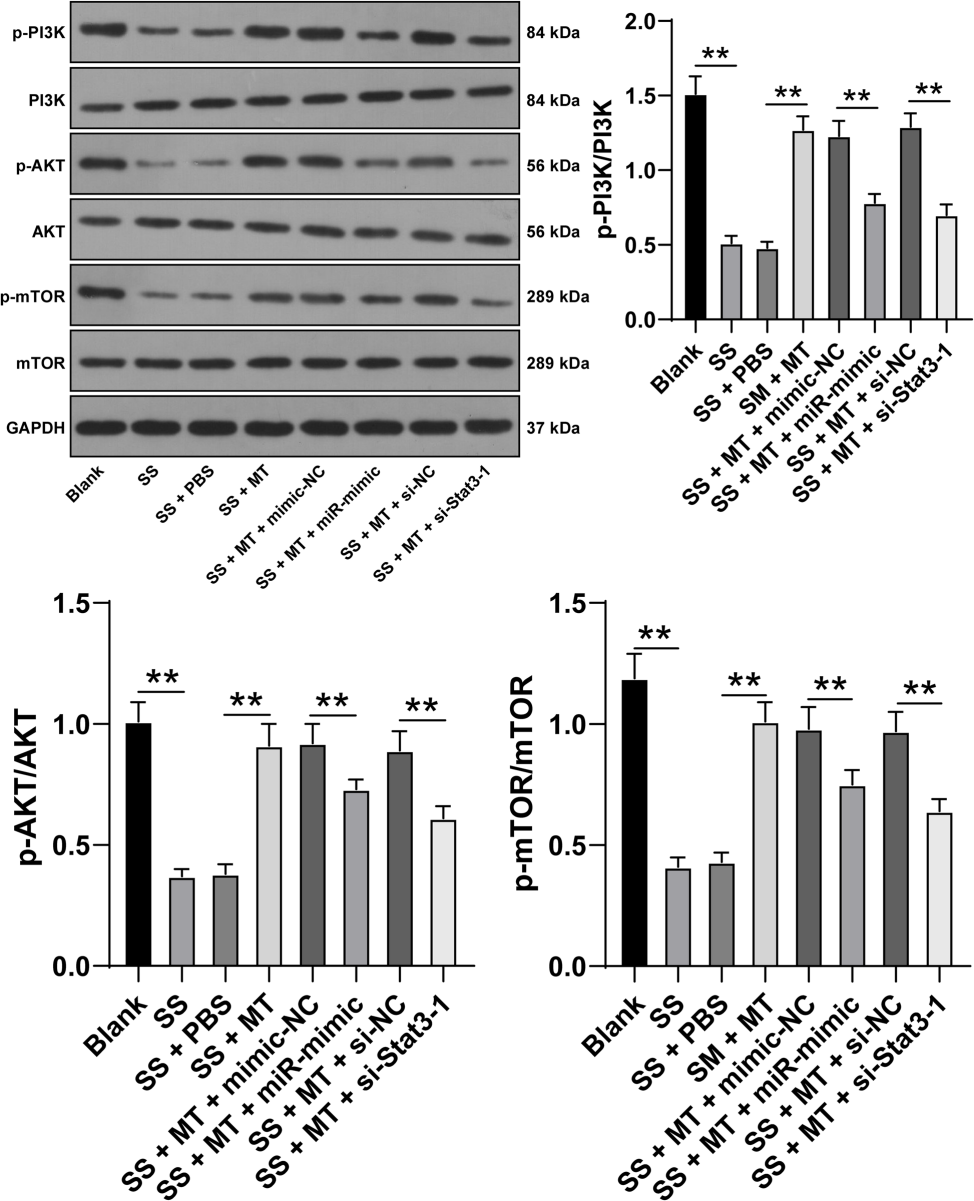
MT activates the PI3K-Akt-mTOR pathway via miR-15a-5p/Stat3. The activation of the PI3K-Akt-mTOR pathway in KGN cells was detected via Western blotting. Experiments were performed in 3 independent repetitions; experimental data were presented as mean ± SD and analyzed via one-way ANOVA, followed by Tukey’s post-hoc test; ** *p* < 0.01

Existing studies have showed that the PI3K-Akt-mTOR pathway is involved in autophagy [31] and plays a role in POI [32]. We determined to verify the speculation that MT may regulate the PI3K-Akt-mTOR pathway via modulating miR-15a-5p/Stat3. The subsequent results revealed that the protein levels of p-PI3K/PI3K, p-Akt/Akt, and p-mTOR/mTOR were decreased in SS-induced KGN cells while increased after MT treatment. Notably, overexpressing miR-15a-5p or silencing Stat3 both decreased the protein levels of p-PI3K/PI3K, p-Akt/Akt, and p-mTOR/mTOR (*p* < 0.05, Fig. 5). Together, these findings indicated that MT activated the PI3K-Akt-mTOR pathway via miR-15a-5p/Stat3.

### Silencing the PI3K-Akt-mTOR pathway promotes SS-induced KGN cell autophagy

**Fig. 6:**
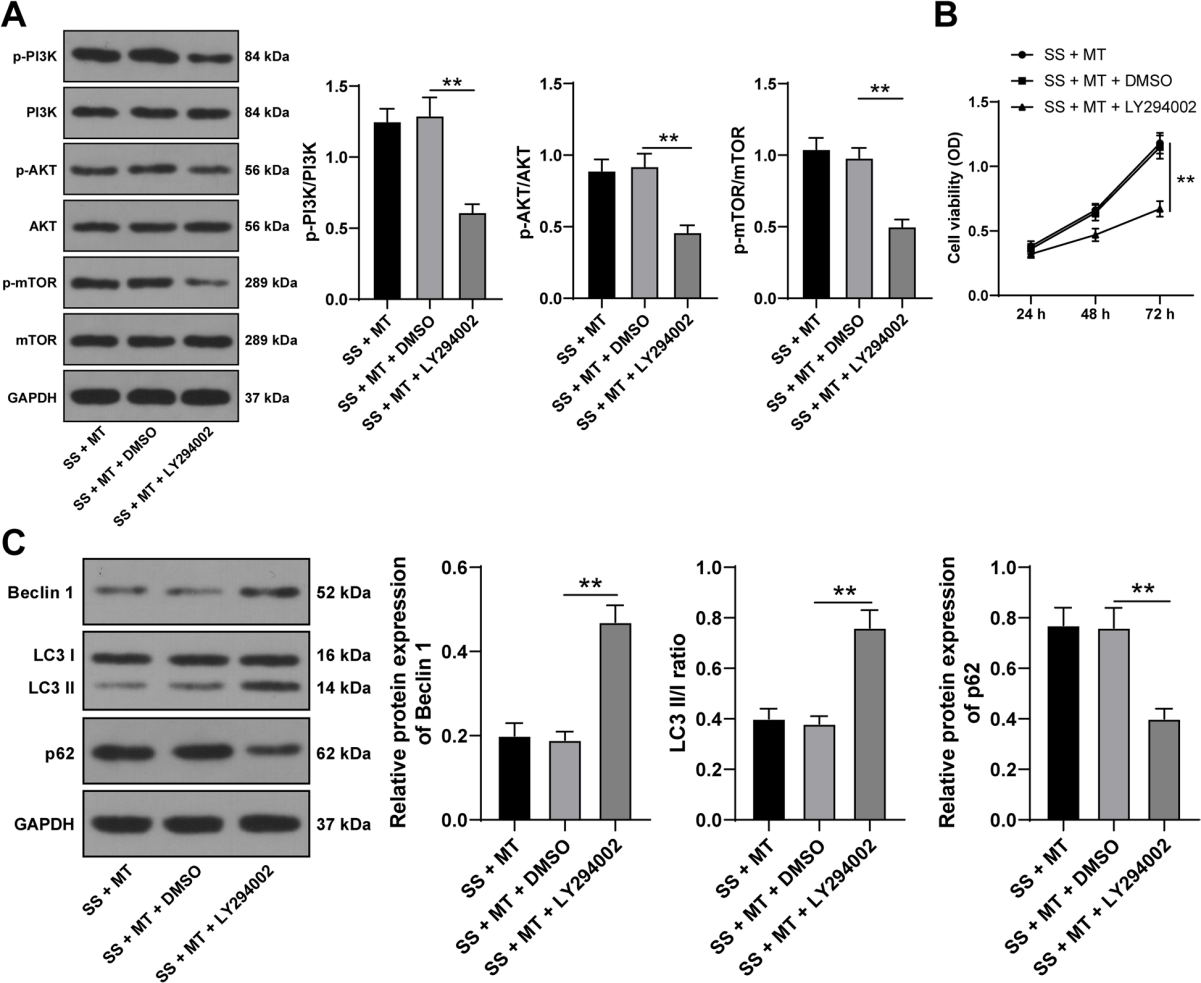
Silencing the PI3K-Akt-mTOR pathway promotes SS-induced KGN cell autophagy. KGN cells were treated with PI3K inhibitor (LY294002) and combined with MT treatment. **A** The activation of the PI3K-Akt-mTOR pathway in KGN cells was detected via Western blotting; **B** KGN cell viability was detected via MTT assay; **C** The expression levels of autophagy-related proteins Beclin 1, LC3 II/I, and p62 in KGN cells were examined via Western blotting. Experiments were performed in 3 independent repetitions; experimental data were presented as mean ± SD; data in figure B were examined via two-way ANOVA and data in figures A and C were examined via one-way ANOVA, followed by Tukey’s post-hoc test; ** *p* < 0.01

Next, to explore whether the PI3K-Akt-mTOR pathway affects the effect of MT on SS-induced KGN cell autophagy, KGN cells were treated with PI3K inhibitor (LY294002) and underwent combined treatment with MT treatment. In the SS + MT + LY294002 group, the protein levels of p-PI3K/PI3K, p-Akt/Akt, and p-mTOR/mTOR in KGN cells were decreased (*p* < 0.05, Fig. 6A), KGN cell viability was reduced (*p* < 0.05, Fig. 6B), Beclin-1 protein levels and LC3 II/I ratio were increased, and p62 protein levels were declined (*p* < 0.05, Fig. 6C). Altogether, the suppression of the PI3K-Akt-mTOR pathway impeded the inhibitory role of MT in SS-induced KGN cell autophagy.

## Discussion

Premature ovarian insufficiency (POI) is termed as a complex and heterogeneous syndrome characterized by irreversibly declined ovarian function before the age of 40 years, and up to now, the fundamental aetiology and the most appropriate management strategy for POI remain to be further explored [33, 34]. Genario et al. has concluded that melatonin (MT) improves oocyte quality and fertility rate, imparting a substantial role in female reproductive system [35]. Additionally, the activation of PI3K-Akt-mTOR pathway is evidenced to exert a protective effect on GC autophagy in PCOS [36]. Herein, our results highlighted that MT regulated GC autophagy in POI through activating the PI3K-Akt-mTOR pathway via the miR-15a-5p/Stat3 axis.

MT possesses anti-inflammatory and anti-oxidative properties and attenuates deoxynivalenol-induced toxicity in GCs in mice [37], and MT also promotes the antioxidant capacity of the ovary of female animals [38]. In addition, MT rescues mitochondrial and ovarian reserve deficiencies induced by excessive autophagy in ovary cells of Chinese hamsters via extracellular signal regulated kinase (ERK) pathway [39]. In this study, KGN cells underwent serum starvation (SS) for establishing GC autophagy models. We found that SS treatment reduced cell viability, elevated Beclin-1 protein levels and LC3 II/I ratio, and decreased p62 protein levels in KGN cells, while MT treatment presented the opposite results. In agreement with our findings, a relevant study has illustrated that MT suppresses autophagic cell death and further mitigates oxidative damage in H_2_O_2_-incubated GCs [40]. On the other hand, MT is proven to alter the biogenesis, intracellular trafficking, and abscission of cells-isolated exosomes at the molecular levels by engaging different effectors [41]. For instance, MT is potent to alter the size and production of exosomes secreted bovine cumulus cells in a dose-dependent way [42], hinting at the effect of MT on paracrine activity of GCs which may further affects GC autophagy. Hence, we concluded that MT can suppress SS-induced KGN cell autophagy.

MT has been shown to modulate noncoding RNAs, including miRNAs, in a variety of diseases and conditions [43, 44]. Existing studies have clarified that overexpression of miR-15a facilitates autophagic activity and increases autophagosomes in Hela cells expressing green fluorescent protein-LC3 [28], and miR-15a induced by tripterygium glycosides affects the progression of POI through modulating GC senescence and cytotoxicity [29], and moreover, miR-15a-5p upregulation is correlated with poor ovarian response [17]. In this study, miR-15a-5p was observed to be up-regulated in the serum of POI patients SS-induced KGN cells and declined in response to MT treatment, and miR-15a-5p upregulation accelerated KGN cell autophagy. Consistently, Wang et al. reported that miR-15a upregulation declines p62 level, activates the Wnt/β-catenin pathway, and further induces autophagy in ammonia-exposed broilers jejunum [45], which supported the results we obtained that miR-15a-5p overexpression counteracted the inhibitory role of MT in SS-induced KGN cell autophagy.

Existing studies have illustrated that Stat3 is closely related to GC and adipocyte proliferation in PCOS [46, 47], and is down-regulated in cisplatin-treated mouse GCs of POI [30]. Moreover, MT activates the JAK2/Stat3 pathway through increasing p-Stat3 and p-JAK2 levels in myocardial ischemia/reperfusion [48], and the binding relation between miR-15a-5p and Stat3 in human umbilical vein fusion cells is also confirmed by Li et al. [49]. In our study, we confirmed the binding of miR-15a-5p and Stat3 and that MT increased Stat3 expression while miR-15a-5p overexpression downregulated Stat3 in KGN cells. Clinically, serum miR-15a-5p expression was negatively correlated to serum Stat3 expression. Furthermore, Stat3 participates in the process of autophagy in a variety of ways [50]. Subsequently, SS-treated KGN cells silencing Stat3 was combined with MT treatment, after which the KGN cell viability was reduced and cell autophagy was promoted. Previous study has proved that leukemia inhibitory factor exerts a repressing effect on GC autophagic cell death via stimulating PI3K/Akt and Stat3 pathways and further alleviates follicular atresia [51], further supporting that Stat3 knockdown can counteract the role of MT in SS-induced KGN cell autophagy.

Kuntai capsule and electro-acupuncture are proven to modulate POI via regulating the PI3K-Akt-mTOR pathway [52, 53], and the inactivation of this pathway suppresses GC proliferation and facilitates GC apoptosis in PCOS [54]. More importantly, MT declines the autophagy markers in rats with hepatic ischemia/reperfusion injury via activating the PI3K/AKT/mTOR/ULK1 pathway [55], miR-15a-5p upregulation deactivates the PI3K-Akt-mTOR pathway in CD4^+^ T cells [56], and Stat3 knockdown inhibits this pathway and exacerbates cell injury induced by HIN1 [57]. Thereafter, we disclosed that p-PI3K/PI3K, p-AKT/AKT, and p-mTOR/mTOR in SS-treated KGN cells were upregulated after MT treatment and were negatively related to miR-15a-5p overexpression or Stat3 suppression, which proved that MT can activate the PI3K-Akt-mTOR pathway via miR-15a-5p/Stat3. Thereafter, the PI3K-Akt-mTOR pathway in SS-treated KGN cells was repressed, followed by MT treatment. The follow-up tests revealed that the repression of this pathway elevated the autophagic level in KGN cells. Previous studies have expounded that Guizhi Fuling Wan represses GC autophagy in PCOS rats through enhancing the PI3K-Akt-mTOR pathway [36], and MT limits dehydroepiandrosterone-exposed KGN cell autophagy and apoptosis through activating the PI3K-Akt pathway and thereby mitigates PCOS [58], further supporting that the inactivation of the PI3K-Akt-mTOR pathway averted the effects of MT on SS-induced KGN cell autophagy.

To sum up, these obtained results initially uncovered that MT limited miR-15a-5p expression to promote Stat3 expression, which activated the PI3K-Akt-mTOR pathway and eventually attenuated SS-induced autophagy in GCs. Excessive autophagy in dysregulated GCs results in follicular atresia, POI, PCOS, and other pathological outcomes [59–61], while this study revealed that MT is potent to inhibit GC autophagy in POI, highlighting the protective benefits of MT in POI-related diseases and providing a solid theoretical basis for the clinical application of MT in the prevention and treatment of adverse gynecological diseases.. However, the clinical sample size we collected in this study was small, and SS-treated KGN cell models cannot fully represent the characteristics POI. Besides, we only conducted this study in vitro, and only investigated the impact of miR-15a-5p/Stat3 axis regulated by MT on the PI3K-Akt-mTOR pathway and failed to explore whether MT can modulate other signaling pathways to affect GC autophagy. Accordingly, in future studies, we will analyze the clinical data on the basis of a larger sample size, verify our conclusions by establishing animal models and other types of cell models, probe other miRs and downstream genes that can be regulated by MT to target the PI3K-Akt-mTOR pathway, explore other autophagy-associated pathways that can be modulated by MT.

## Supplementary Information

**Additional file 1.**

## Data Availability

The datasets used and/or analysed during the current study are available from the corresponding author on reasonable request.
