# Supplementary material for: Melatonin suppresses serum starvation-induced autophagy of ovarian granulosa cells in premature ovarian insufficiency
Source: BMC Womens Health. 2022 Nov 24;22:474. doi: 10.1186/s12905-022-02056-7 (PMC9700896; doi:10.1186/s12905-022-02056-7)
Supplement: Supplementary file 1 — Additional file 1. [file 12905_2022_2056_MOESM1_ESM.pdf]

Unedited blots for Figure 1B

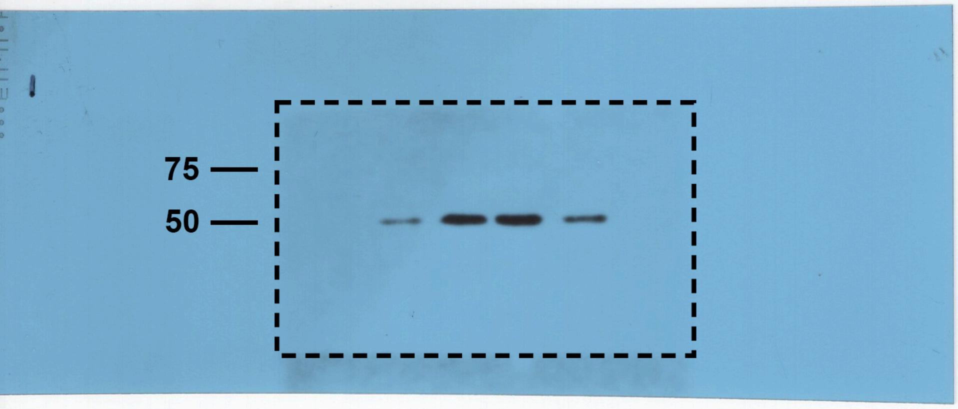

Beclin 1

52 kDa

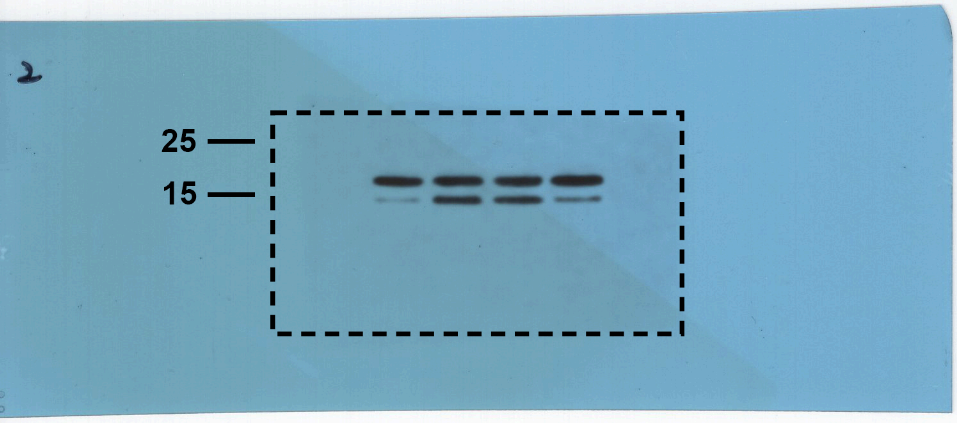

LC3 I
LC3 II

16 kDa
14 kDa

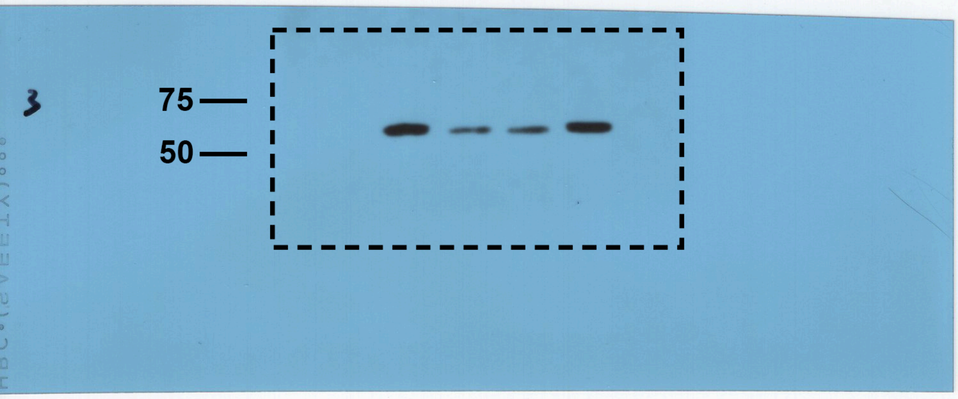

p62

62 kDa

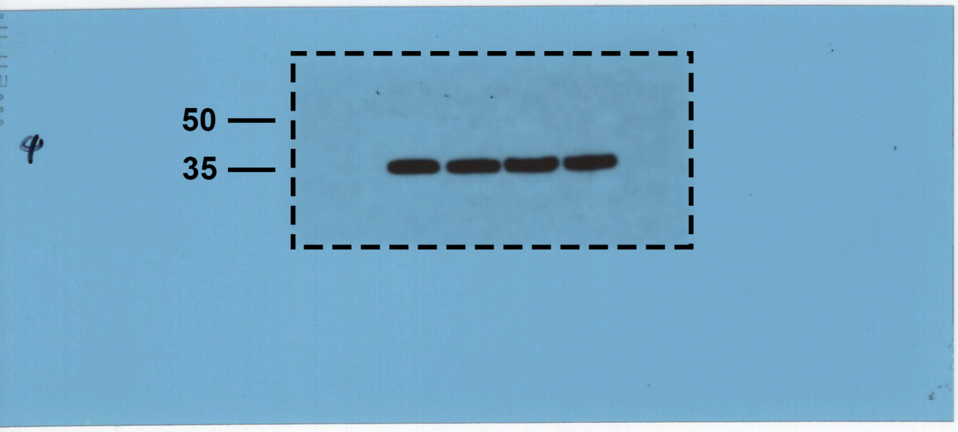

GAPDH

37 kDa

Unedited blots for Figure 2C

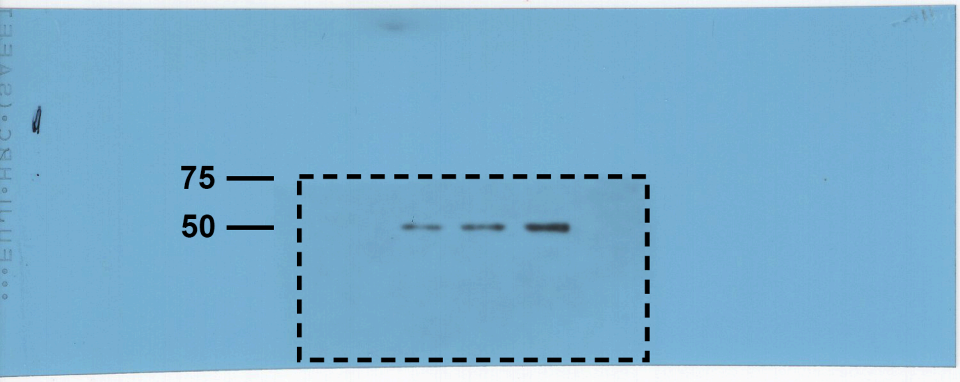

Beclin 1
52 kDa

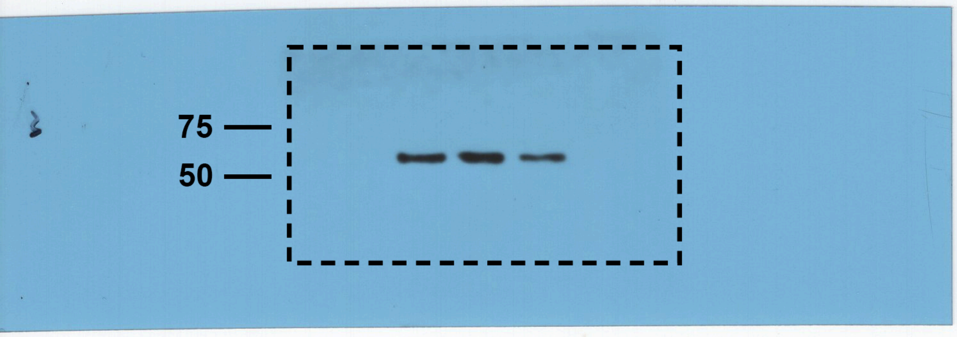

p62
62 kDa

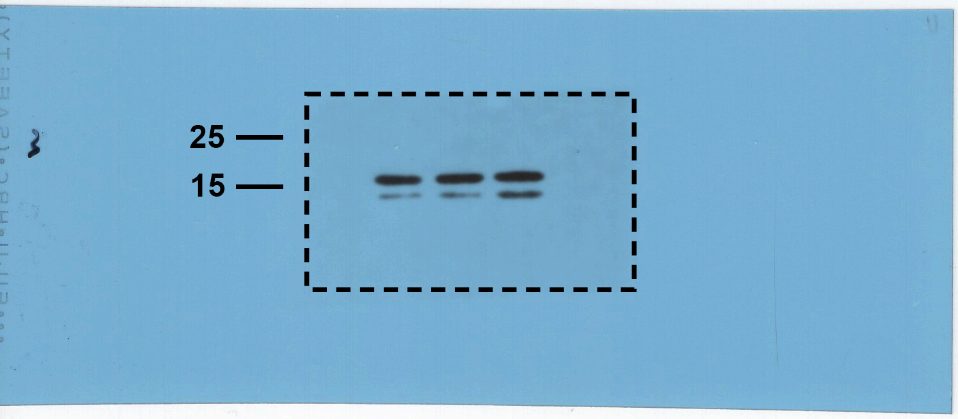

LC3 I
LC3 II
16 kDa
14 kDa

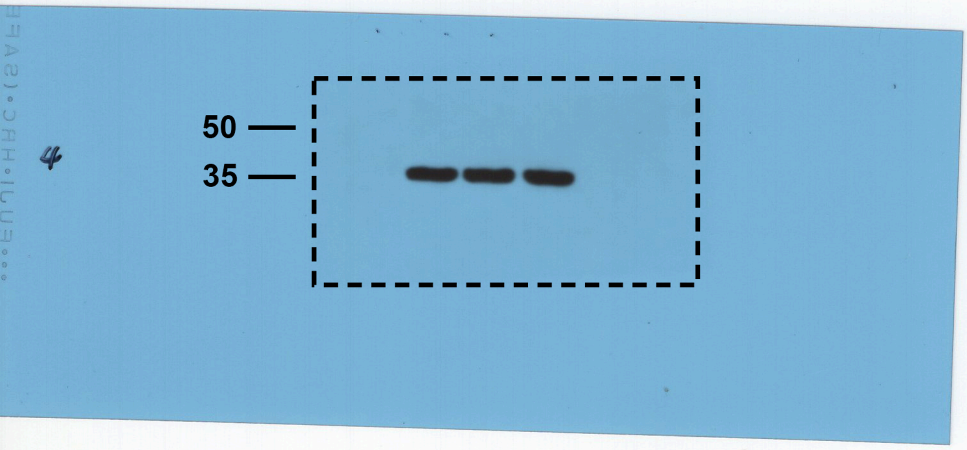

GAPDH
37 kDa

Unedited blots for Figure 3C

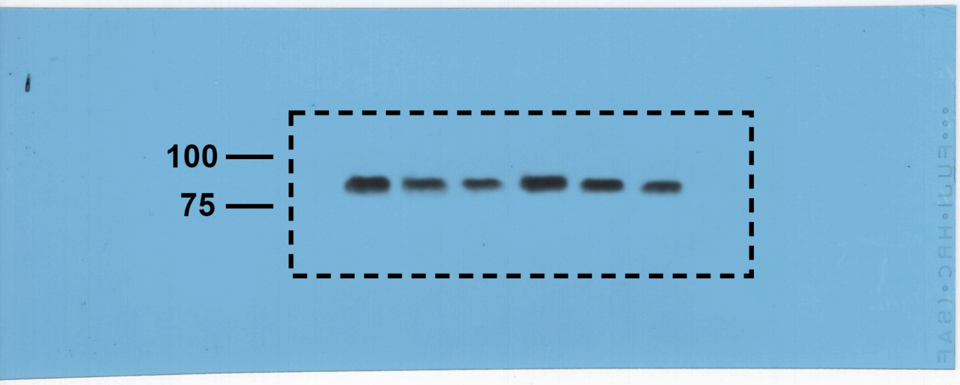

Stat3
88 kDa

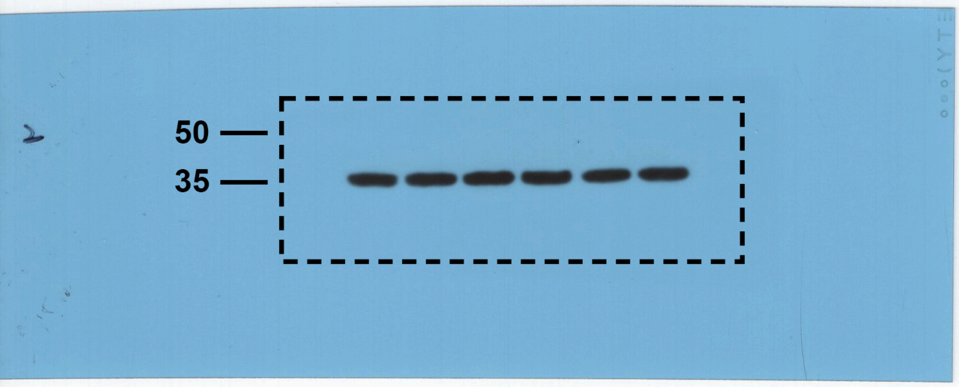

GAPDH
37 kDa

Unedited blots for Figure 4B

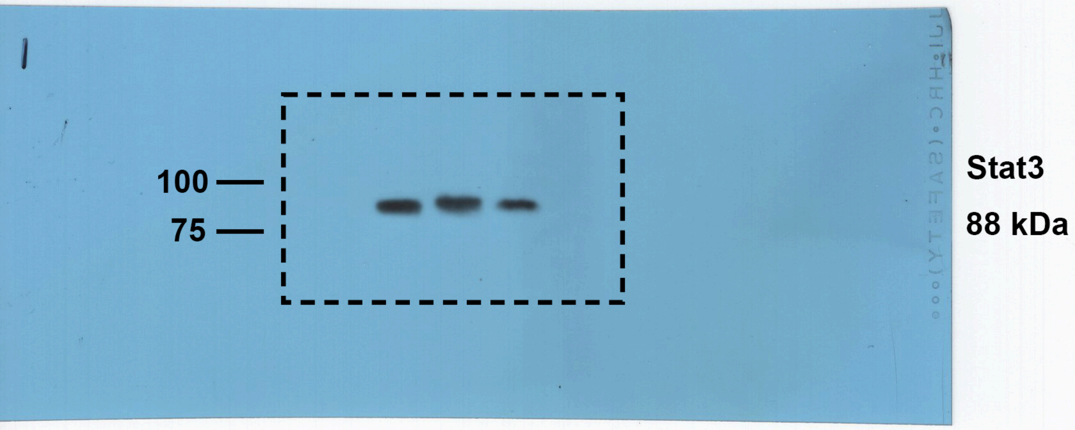

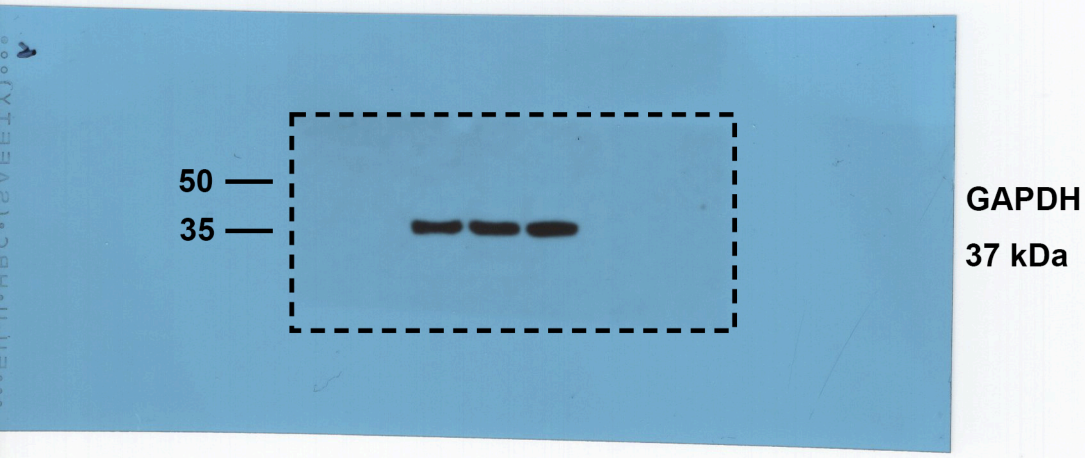

Unedited blots for Figure 4D

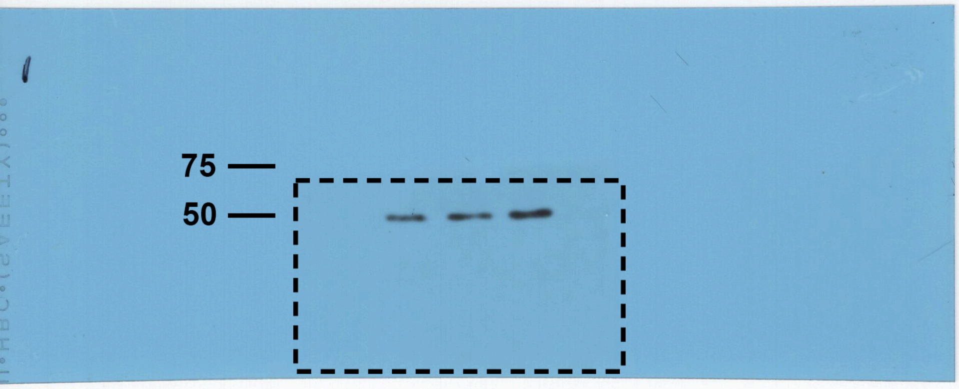

Beclin 1
52 kDa

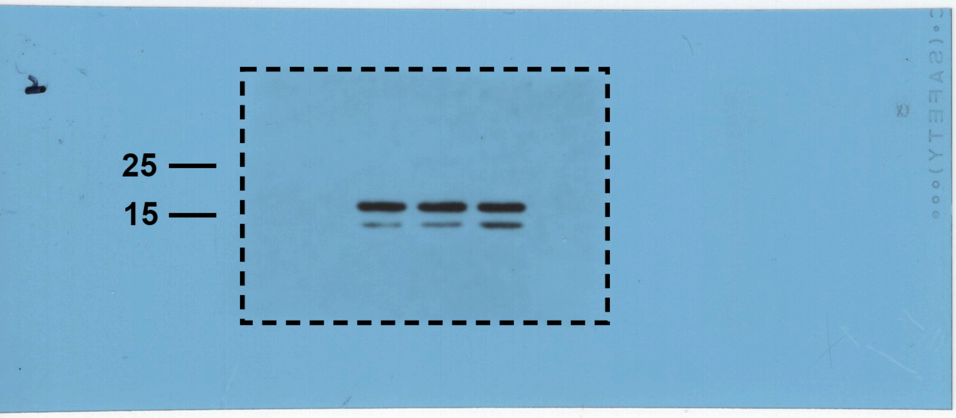

LC3 I
LC3 II
16 kDa
14 kDa

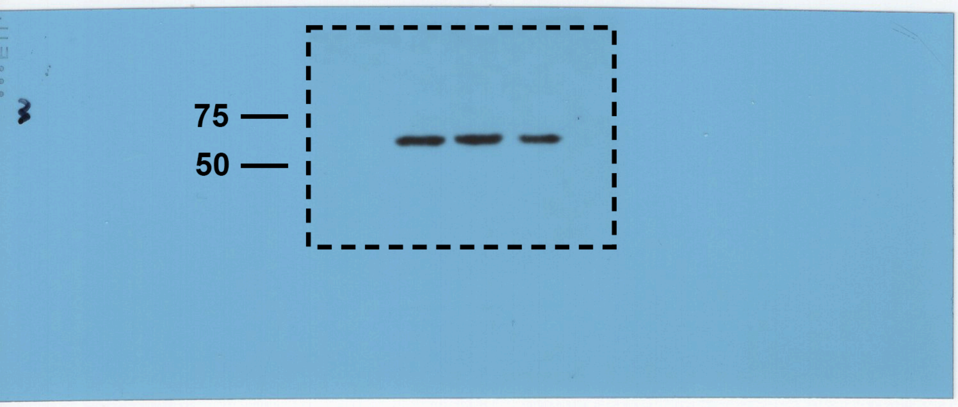

p62
62 kDa

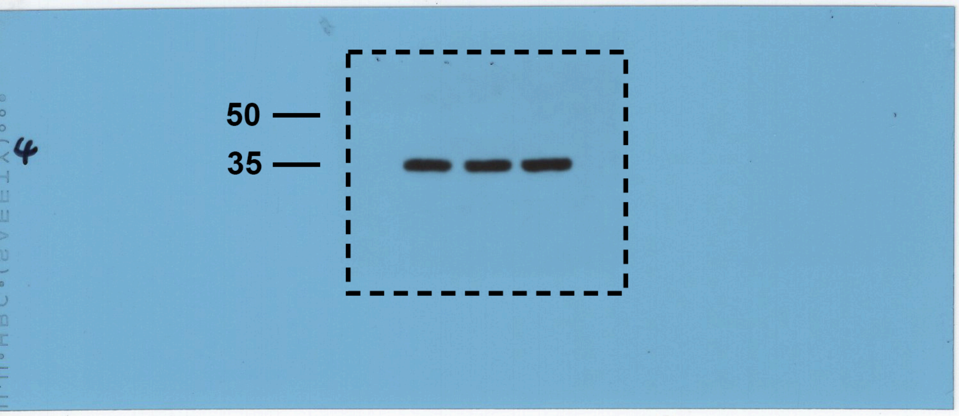

GAPDH
37 kDa

Unedited blots for Figure 5

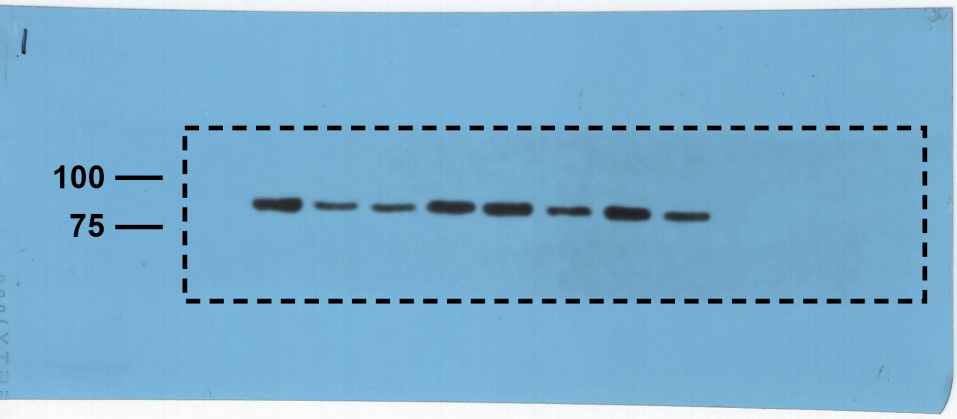

p-PI3K
84 kDa

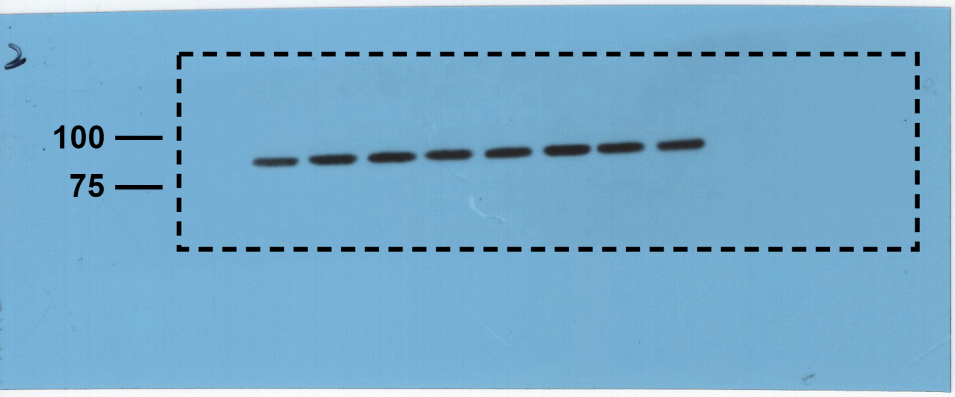

PI3K
84 kDa

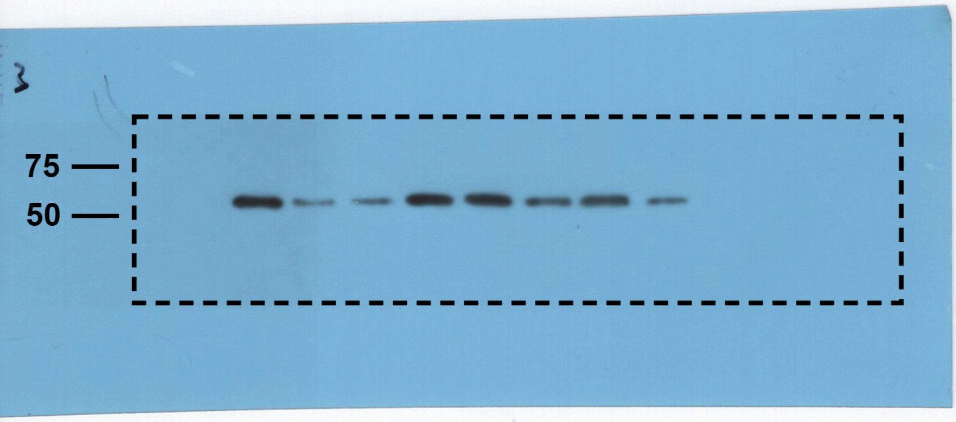

P-AKT
56 kDa

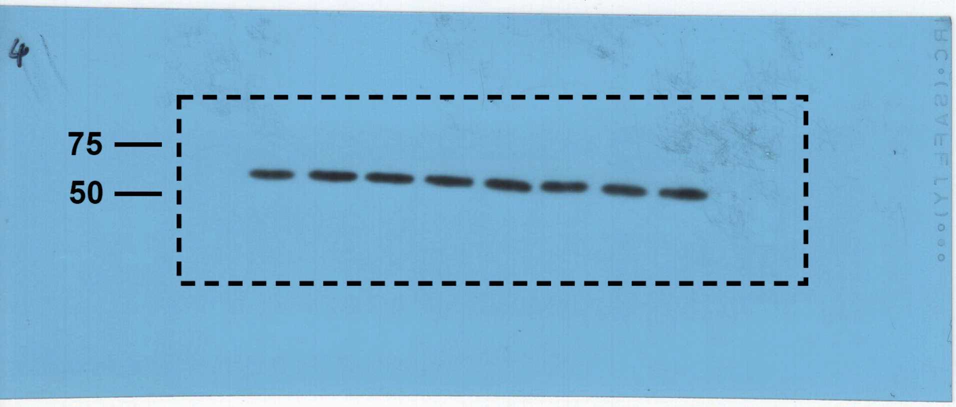

AKT
56 kDa

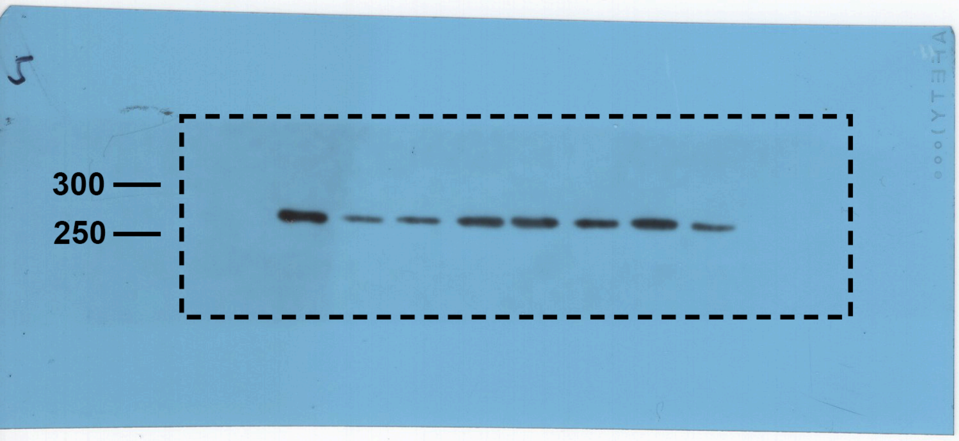
300
250
p-mTOR
289 kDa

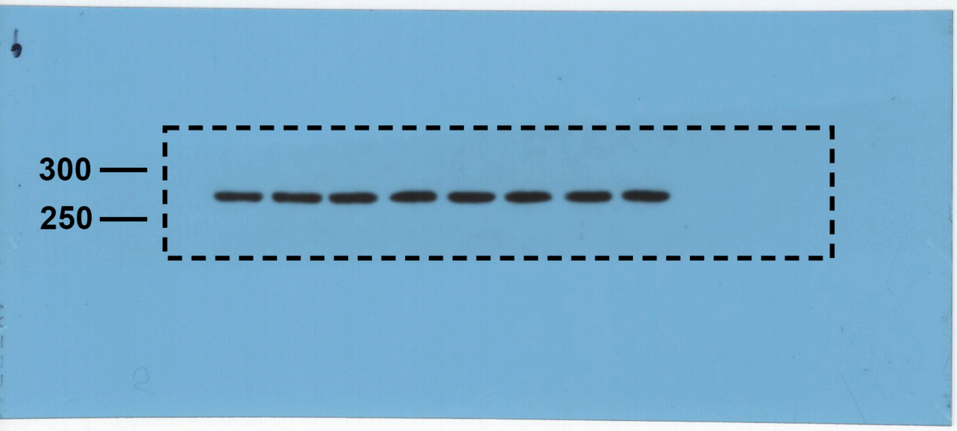
300
250
mTOR
289 kDa

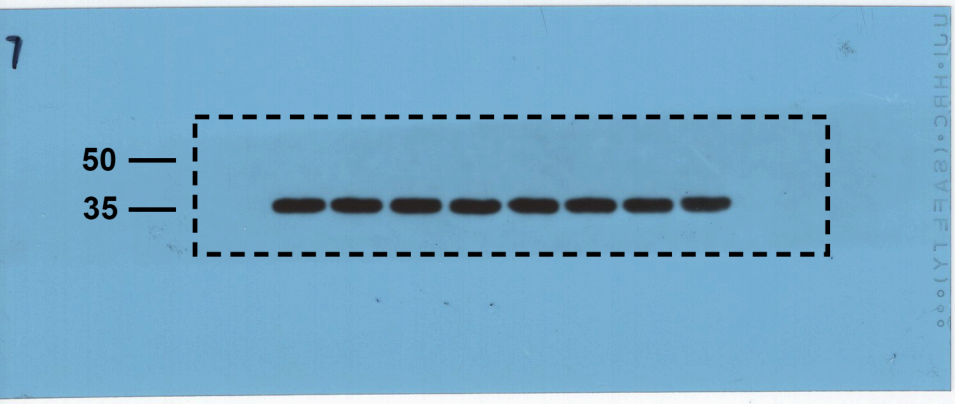
50
35
GAPDH
37 kDa

Unedited blots for Figure 6A

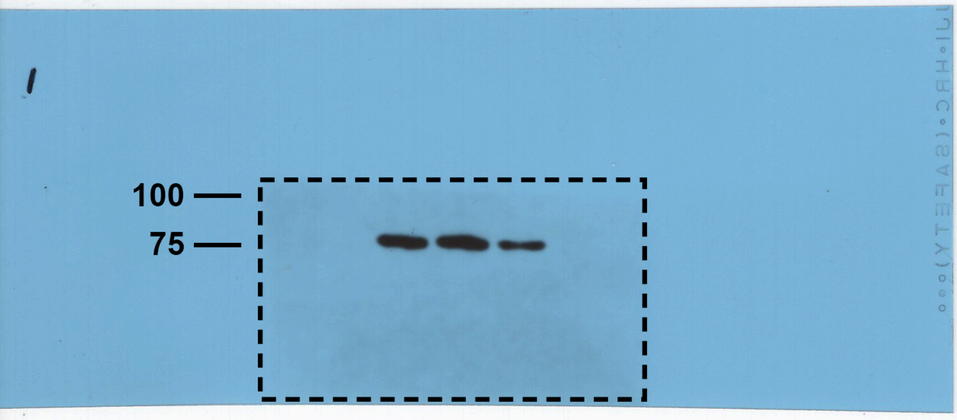

p-PI3K

84 kDa

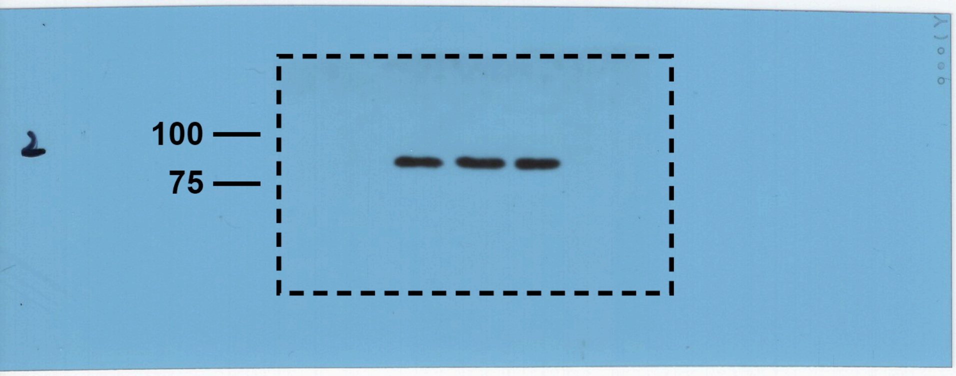

PI3K

84 kDa

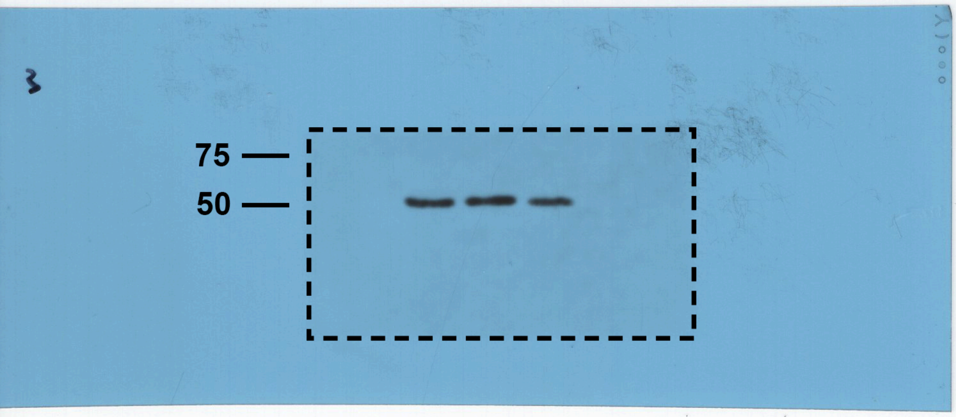

P-AKT

56 kDa

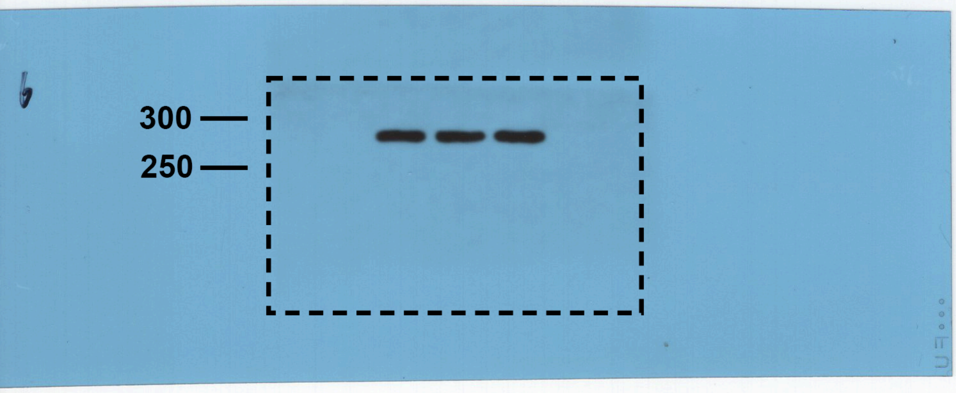

mTOR

289 kDa

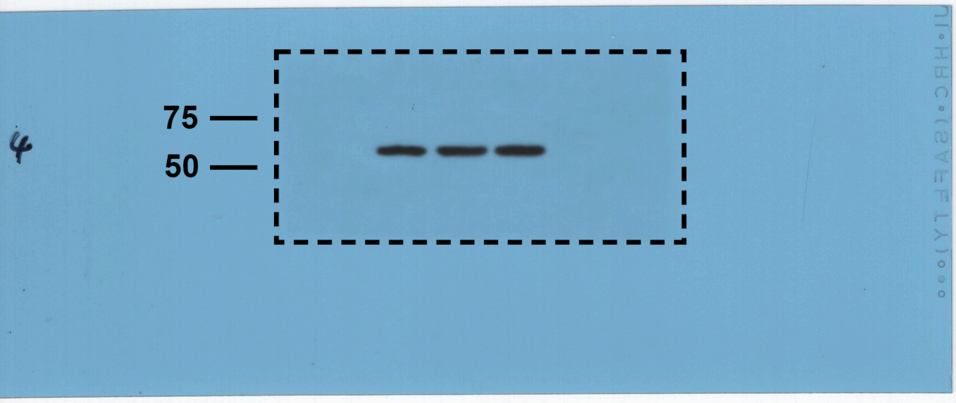

AKT
56 kDa

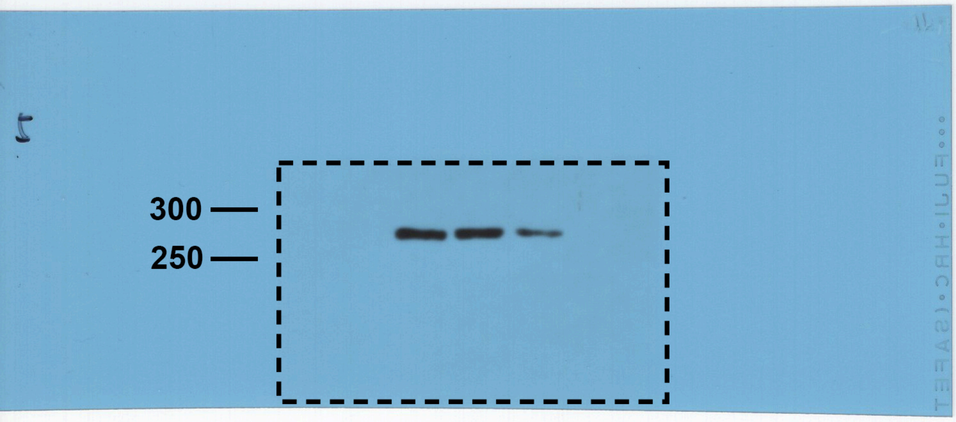

p-mTOR
289 kDa

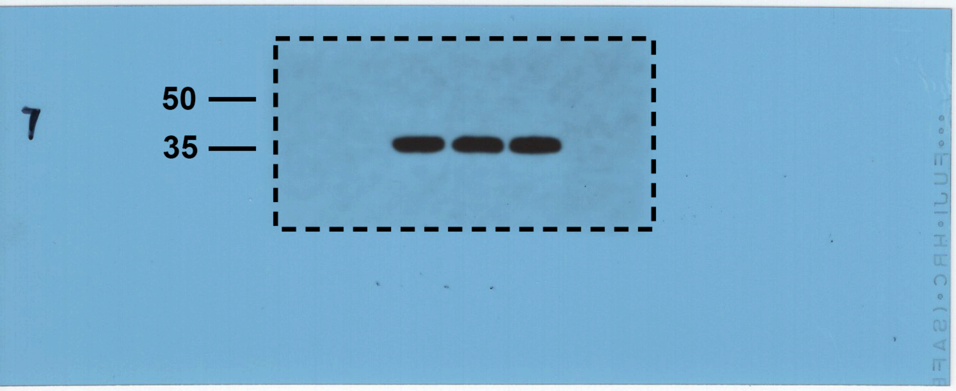

GAPDH
37 kDa

Unedited blots for Figure 6C

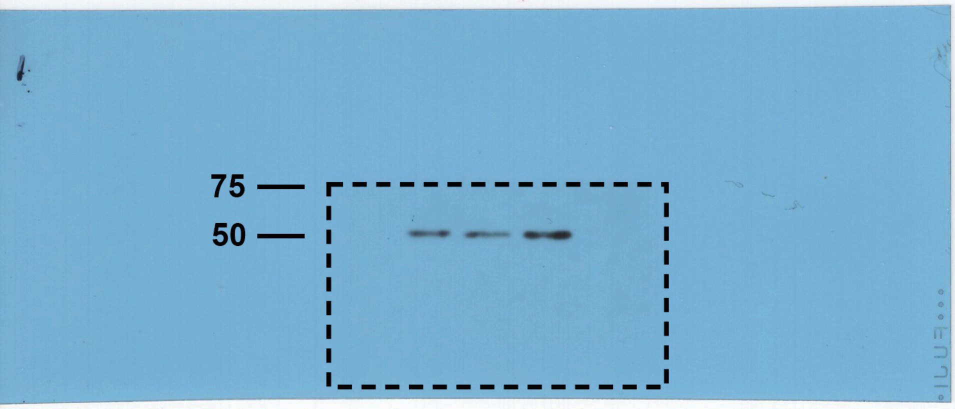

Beclin 1

52 kDa

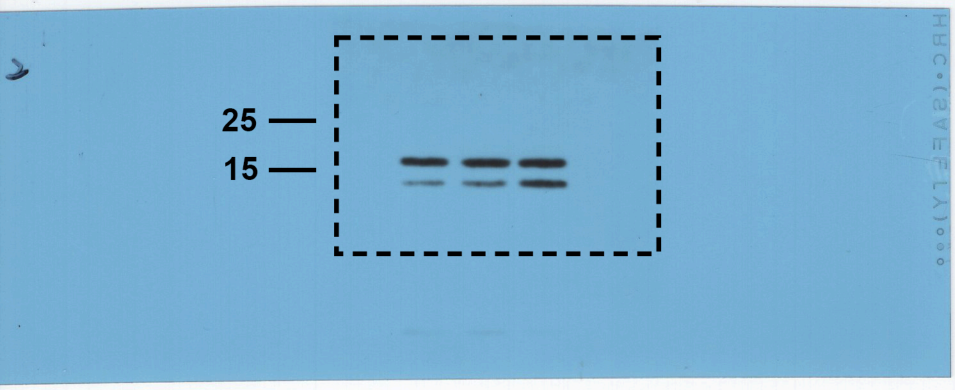

LC3 I
LC3 II

16 kDa
14 kDa

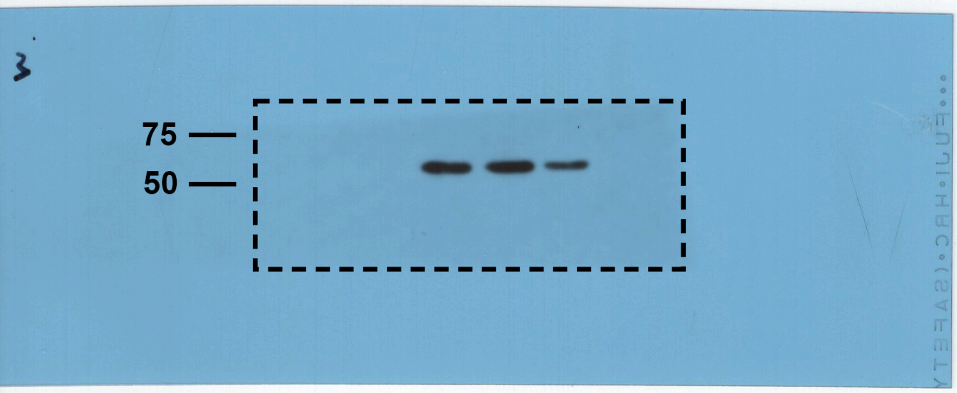

p62

62 kDa

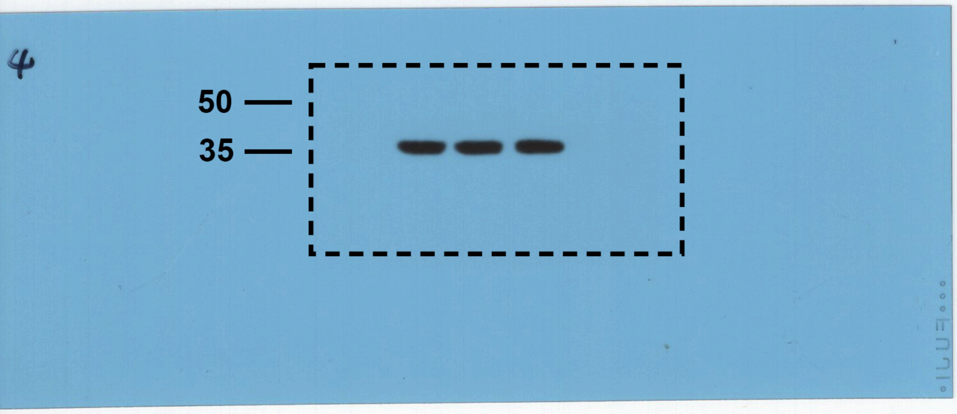

GAPDH

37 kDa

Unedited blots for Figure 1B

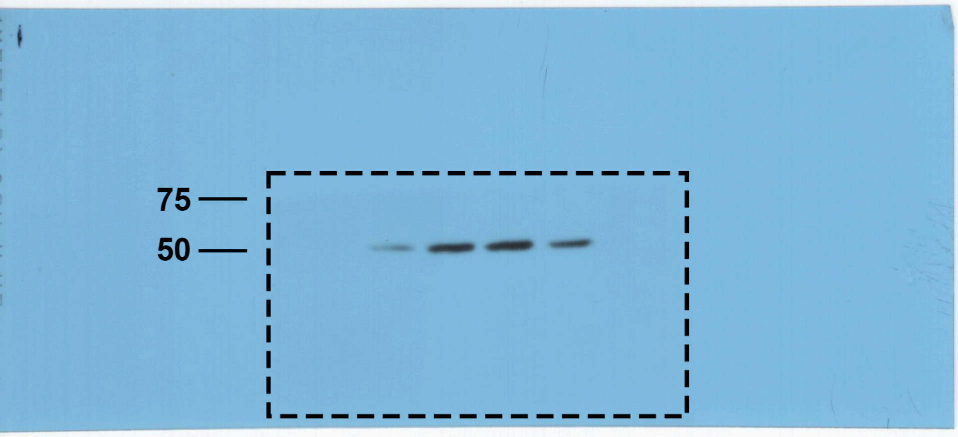

Beclin 1
52 kDa

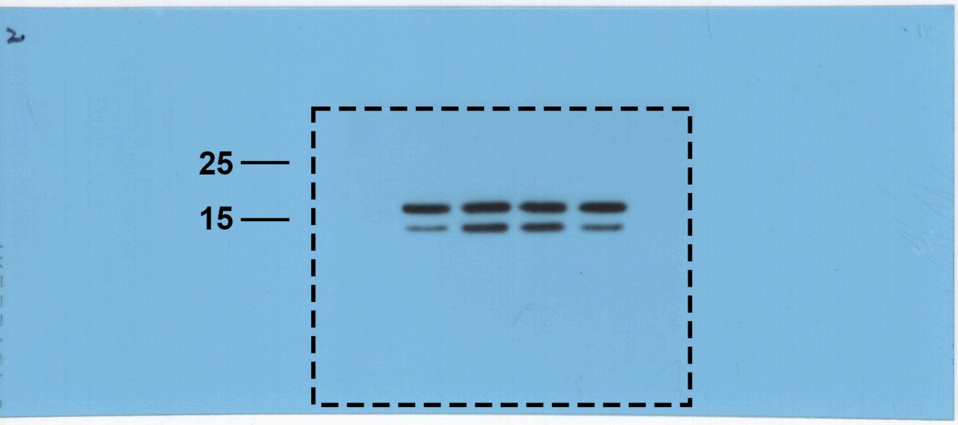

LC3 I
LC3 II
16 kDa
14 kDa

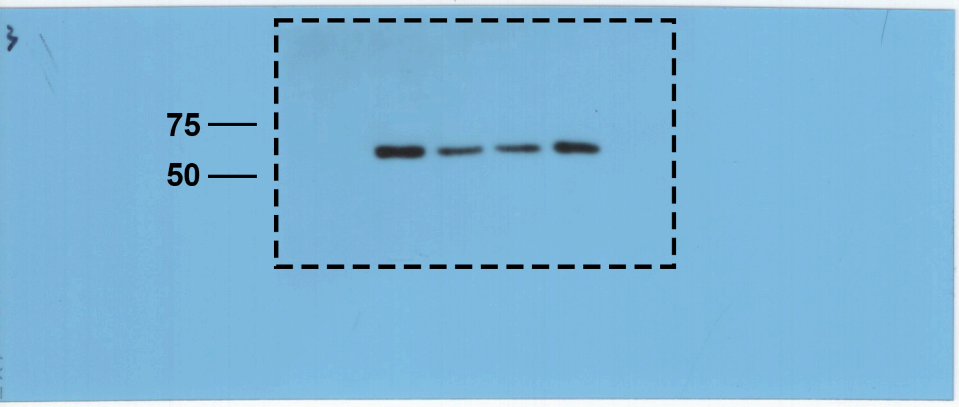

p62
62 kDa

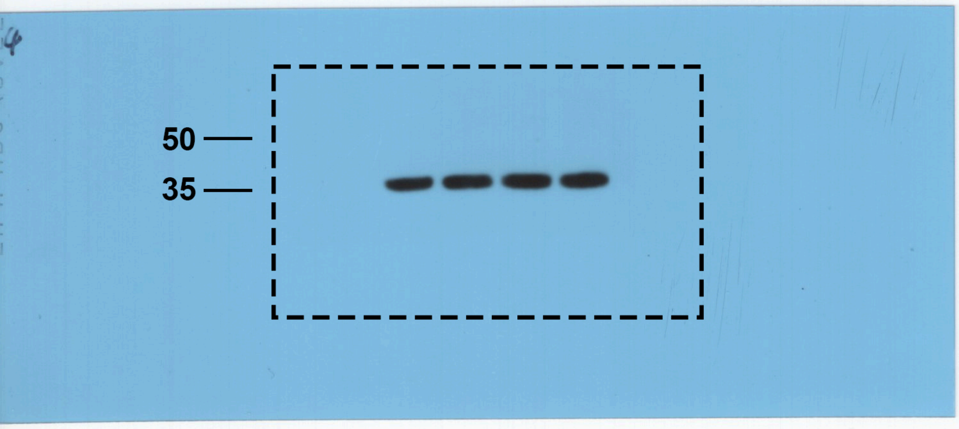

GAPDH
37 kDa

Unedited blots for Figure 2C

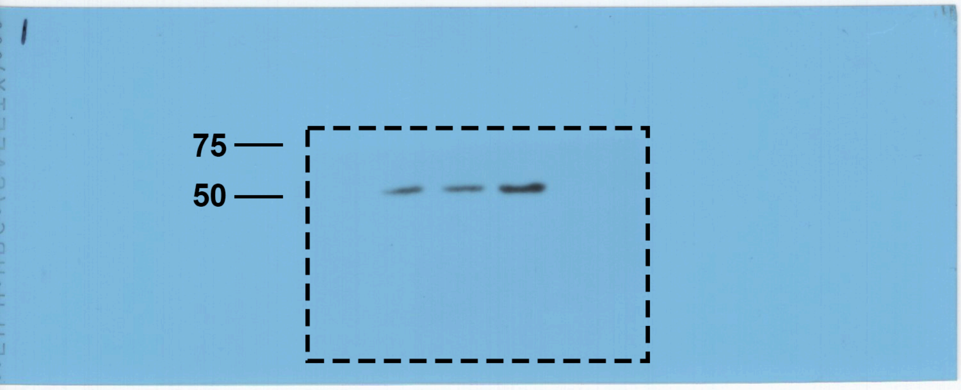

Beclin 1
52 kDa

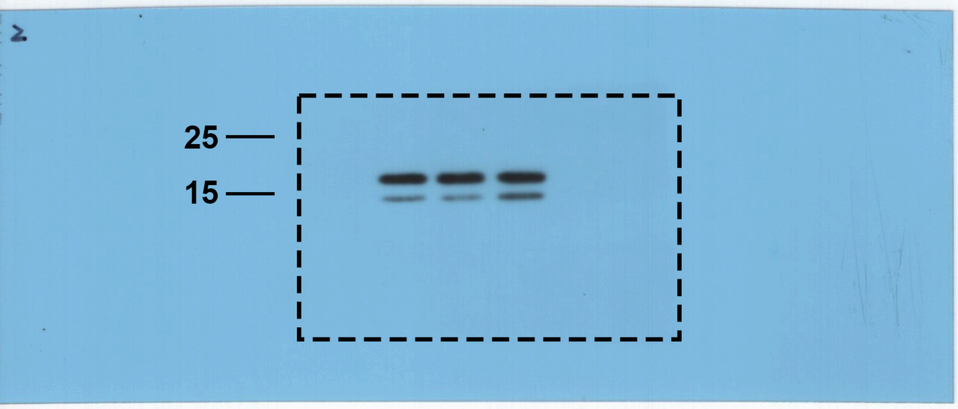

LC3 I
LC3 II
16 kDa
14 kDa

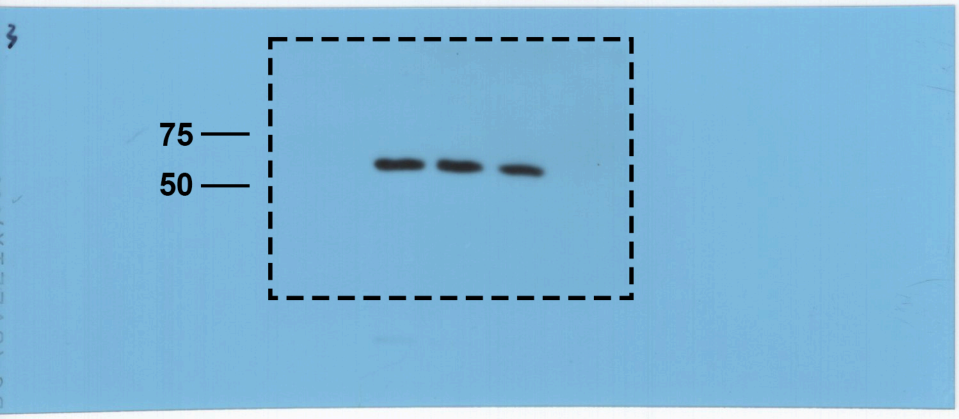

p62
62 kDa

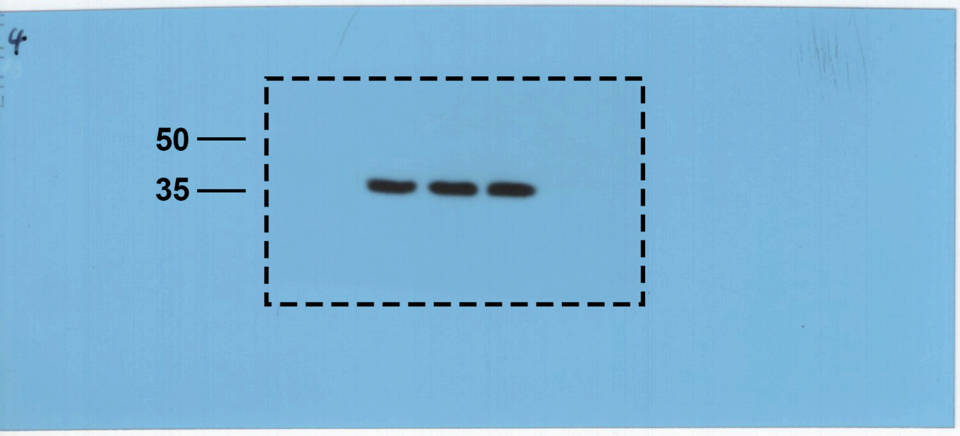

GAPDH
37 kDa

Unedited blots for Figure 3C

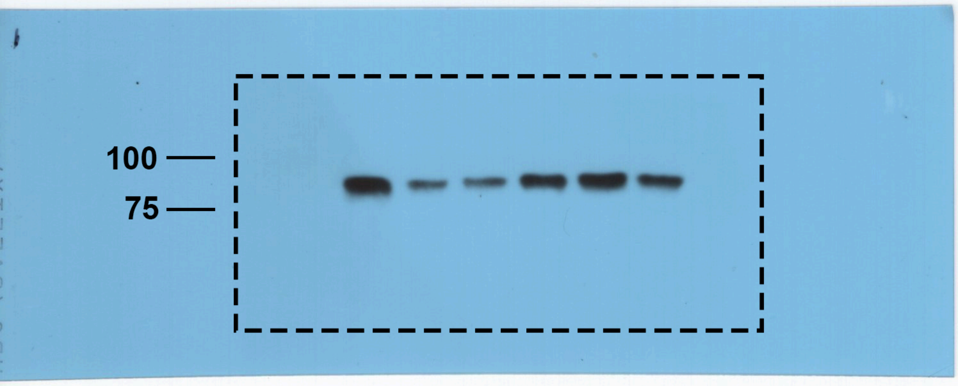

Stat3
88 kDa

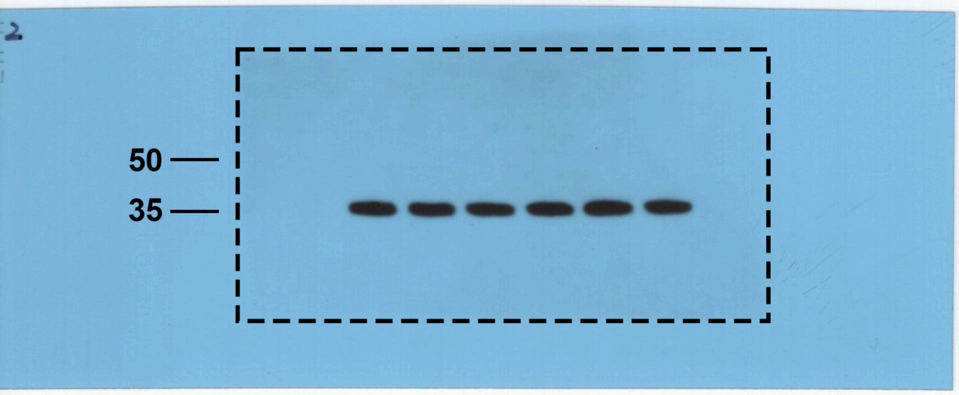

GAPDH
37 kDa

Unedited blots for Figure 4B

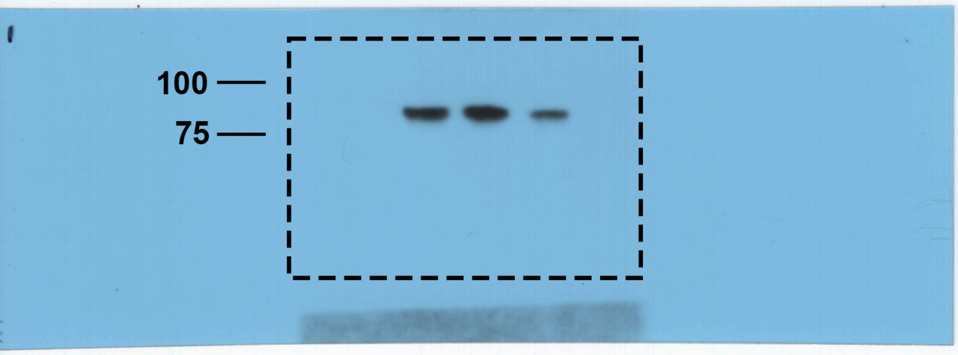

Stat3
88 kDa

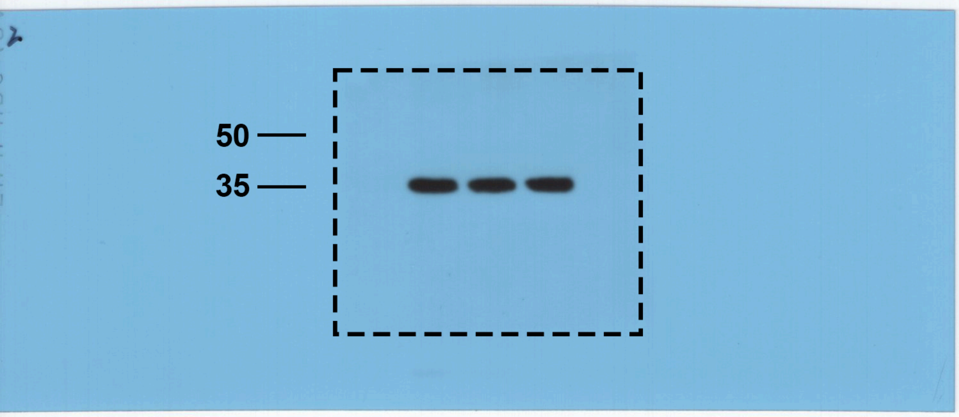

GAPDH
37 kDa

Unedited blots for Figure 4D

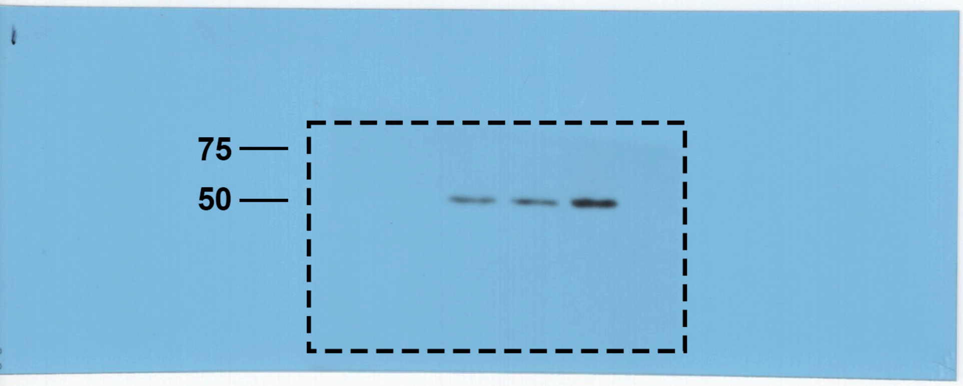

Beclin 1

52 kDa

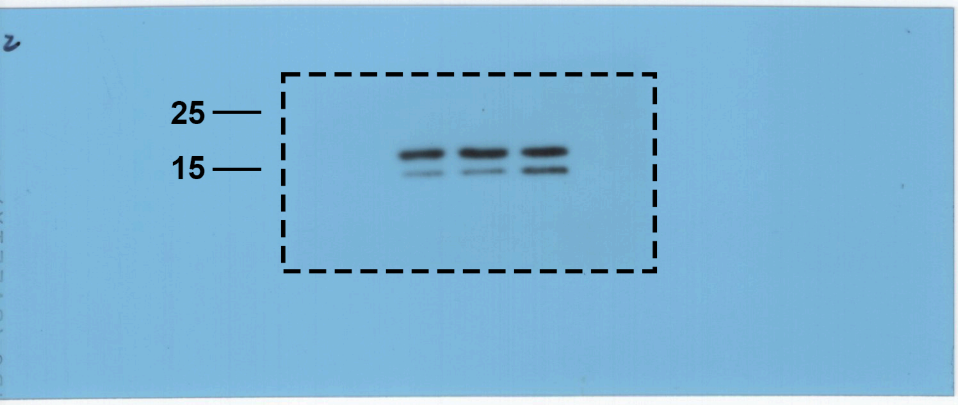

LC3 I

LC3 II

16 kDa

14 kDa

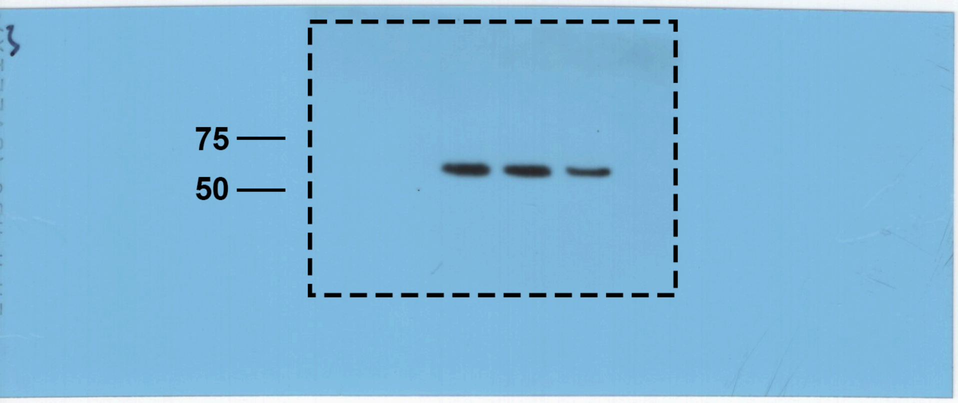

p62

62 kDa

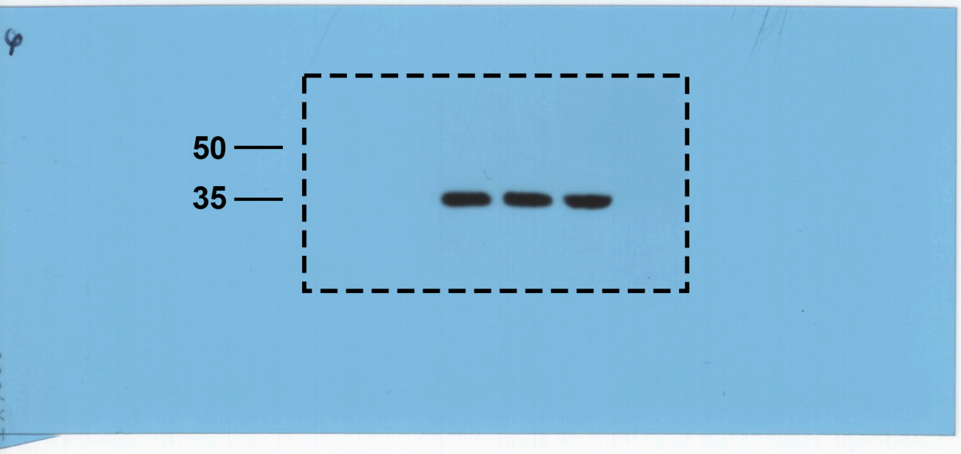

GAPDH

37 kDa

Unedited blots for Figure 5

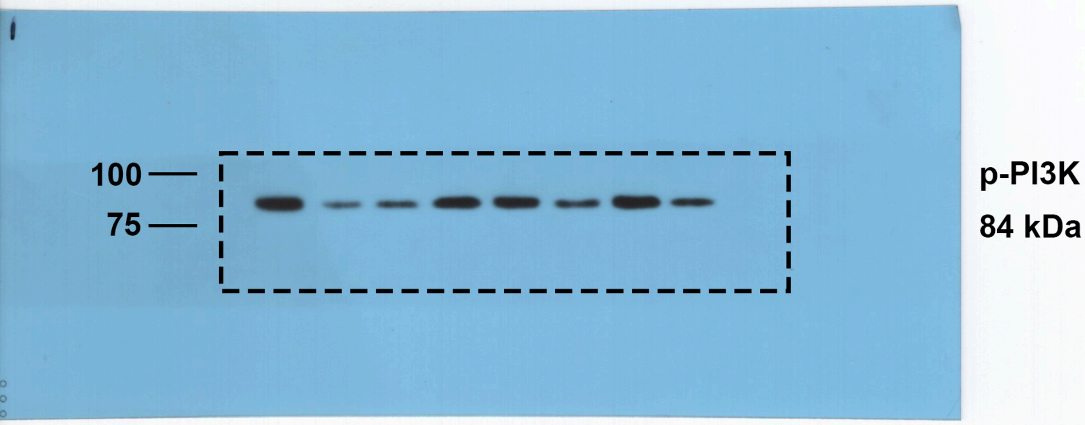

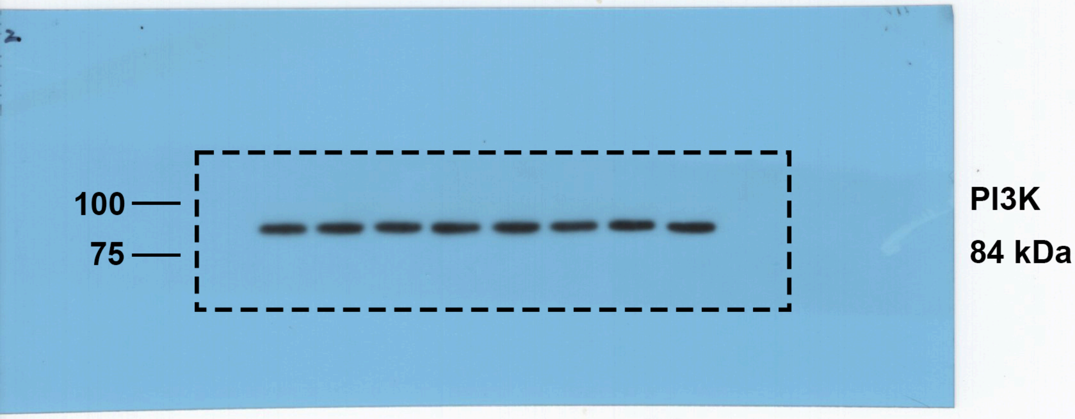

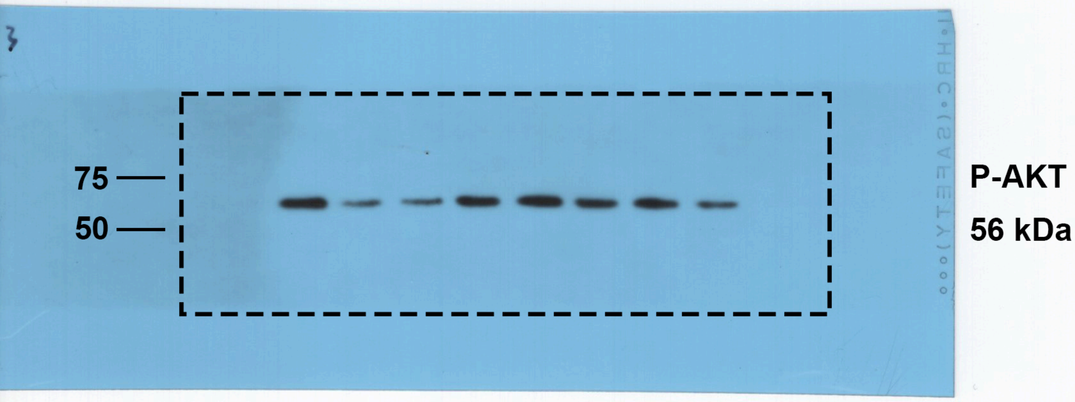

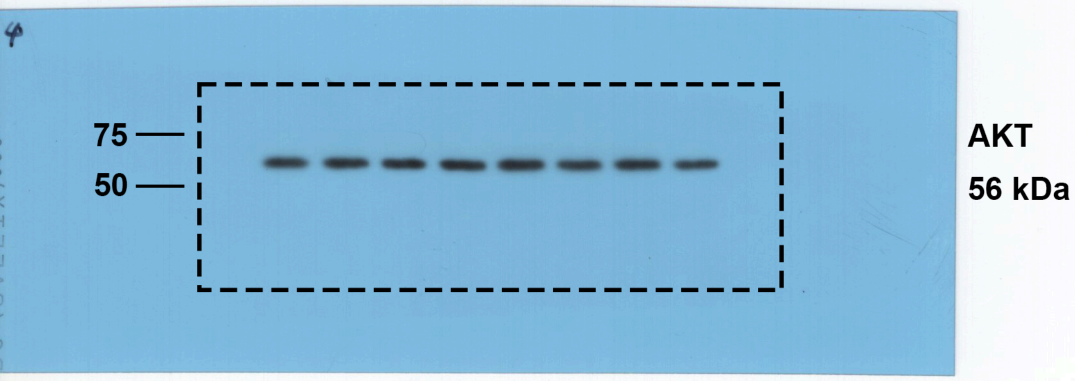

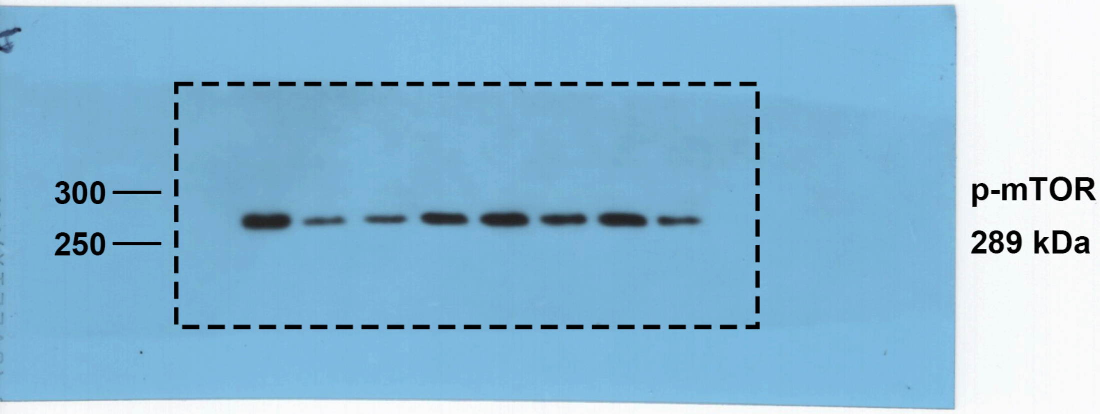
300
250
p-mTOR
289 kDa

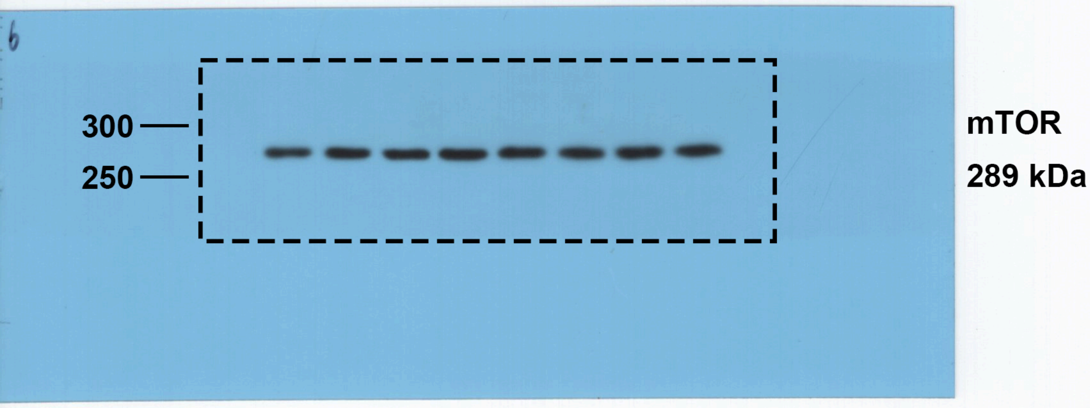
300
250
mTOR
289 kDa

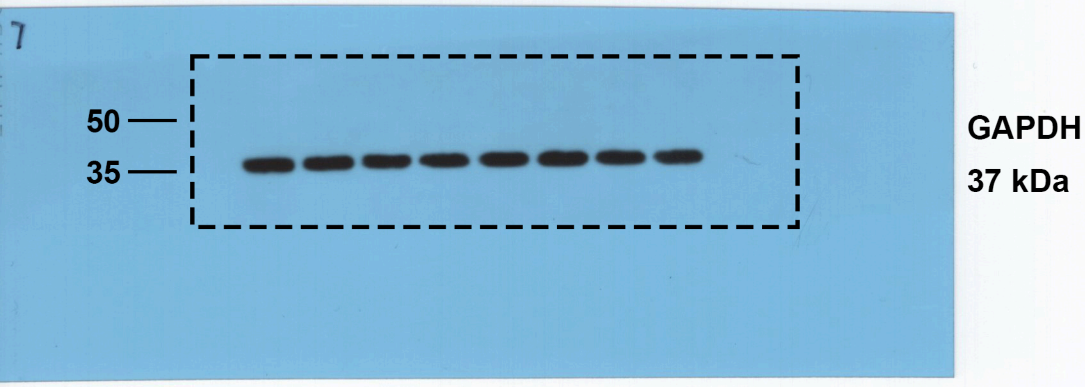
50
35
GAPDH
37 kDa

Unedited blots for Figure 6A

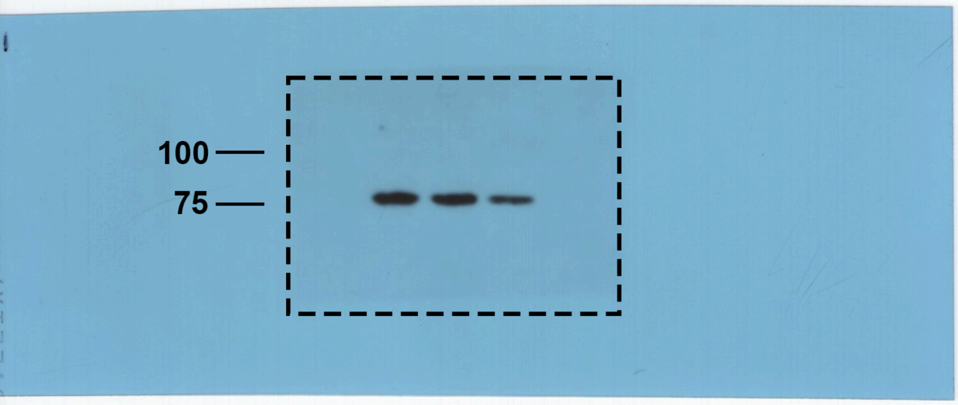

p-PI3K
84 kDa

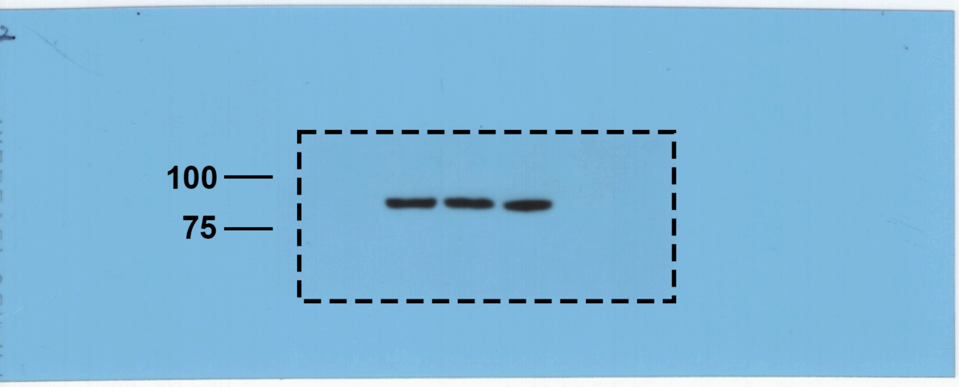

PI3K
84 kDa

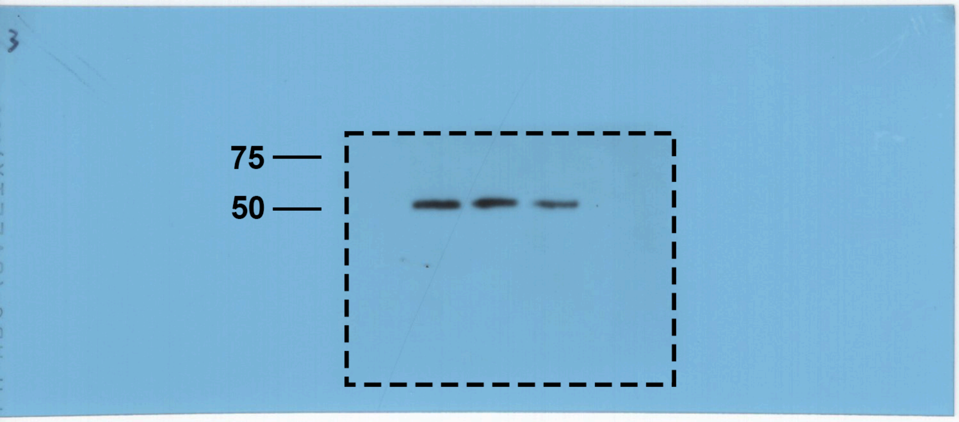

P-AKT
56 kDa

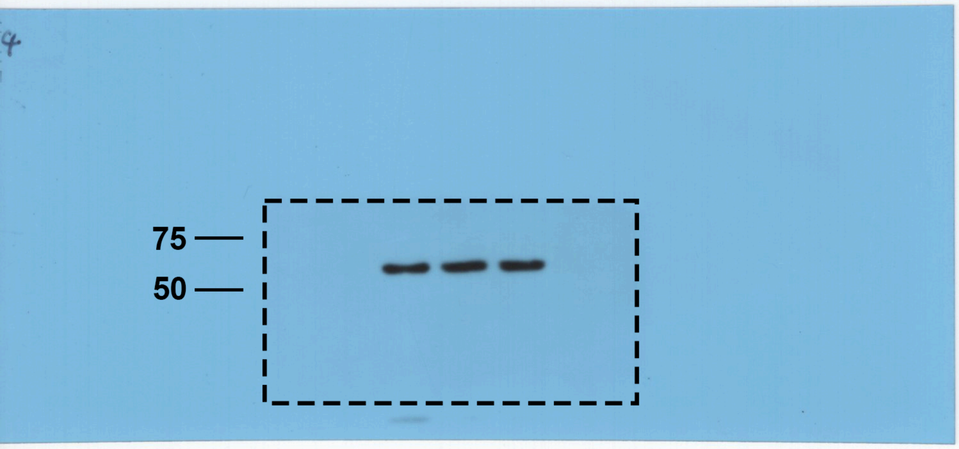

AKT
56 kDa

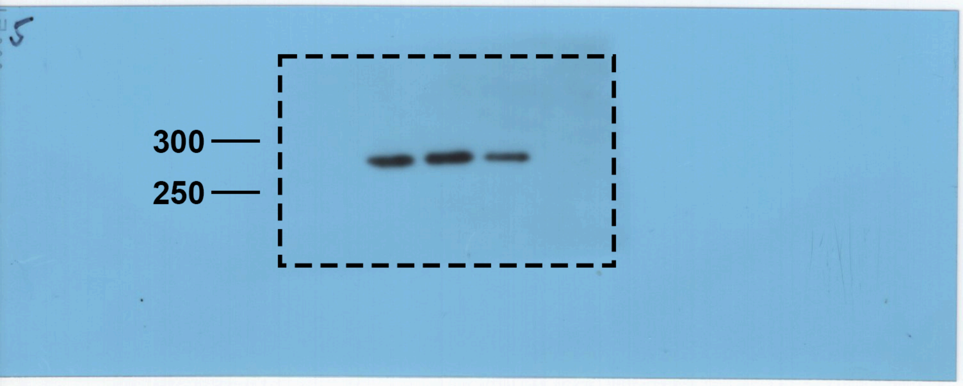

p-mTOR
289 kDa

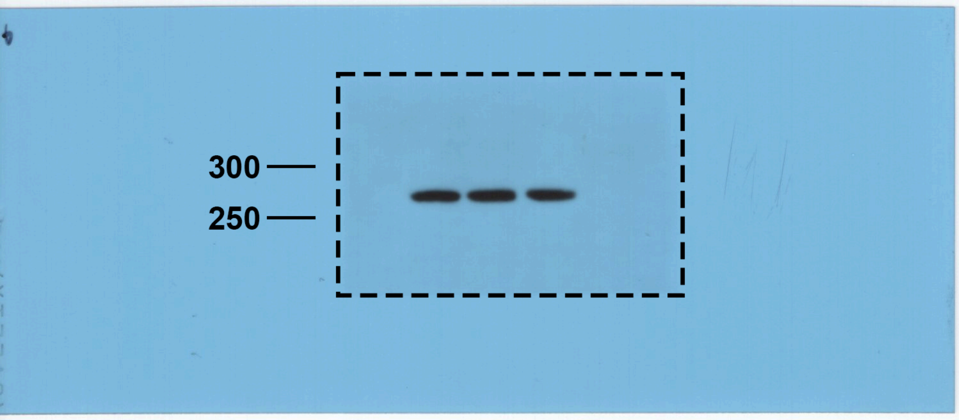

mTOR
289 kDa

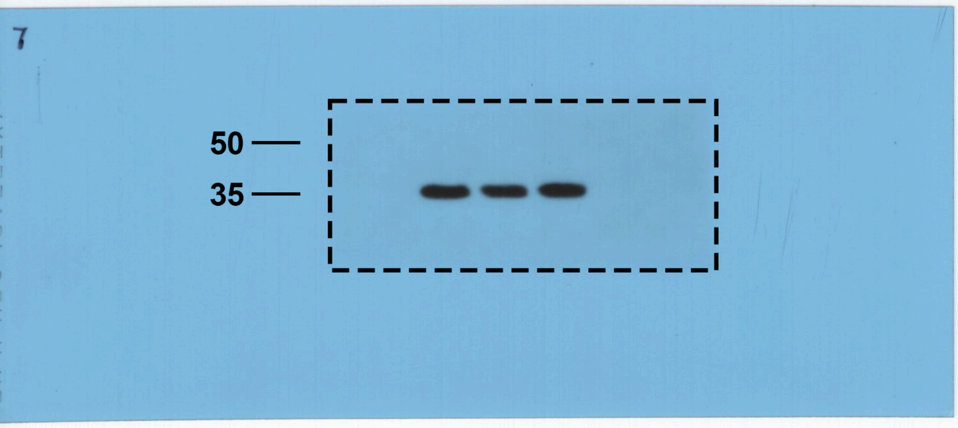

GAPDH
37 kDa

Unedited blots for Figure 6C

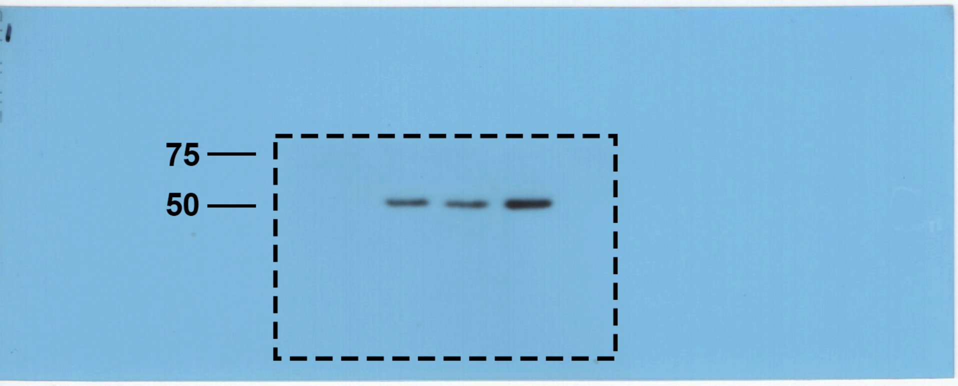

Beclin 1

52 kDa

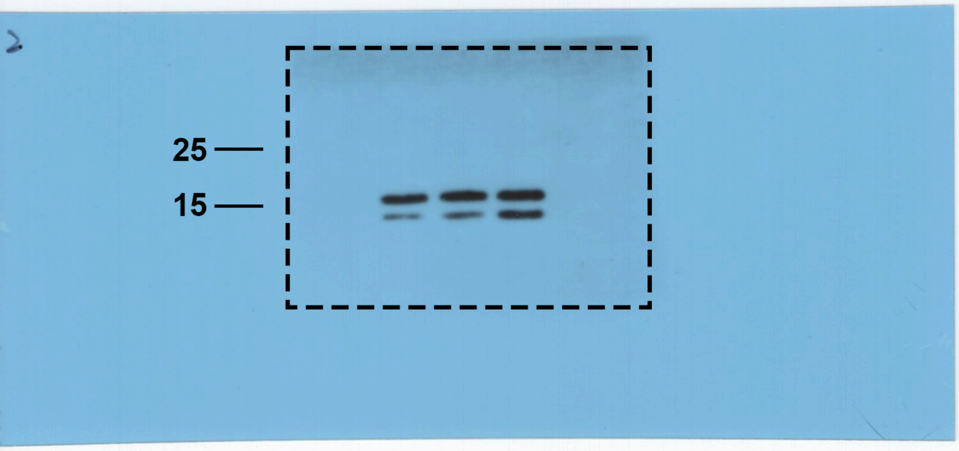

LC3 I

LC3 II

16 kDa

14 kDa

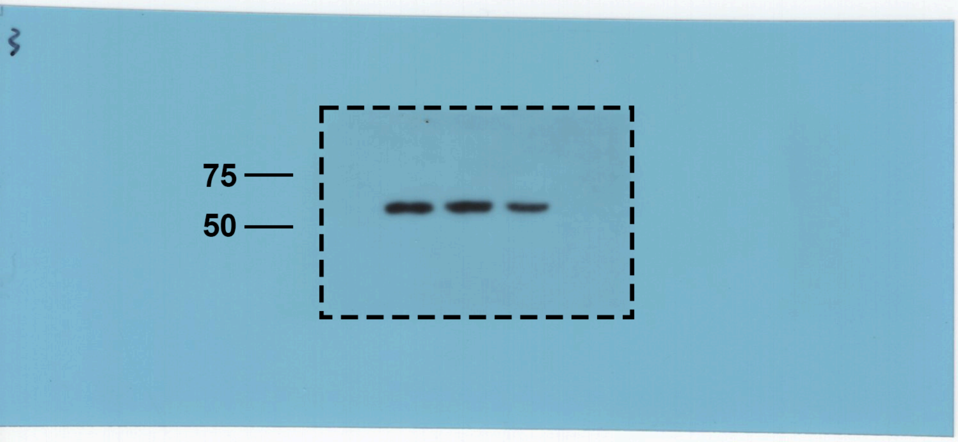

p62

62 kDa

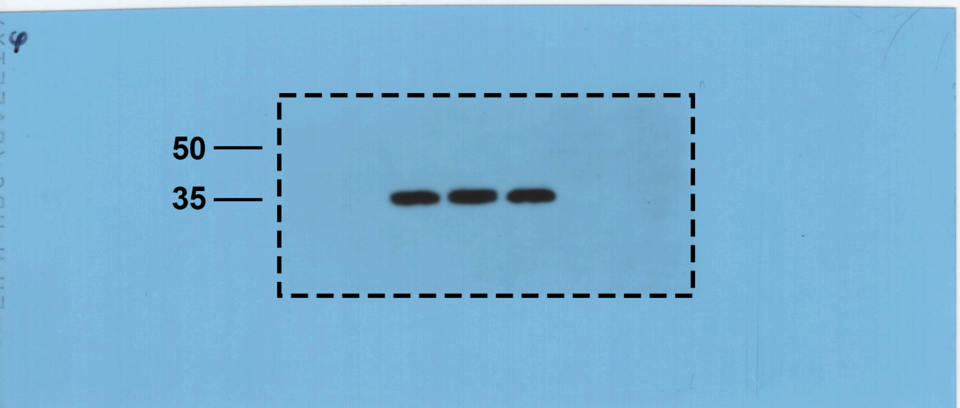

GAPDH

37 kDa

Unedited blots for Figure 1B

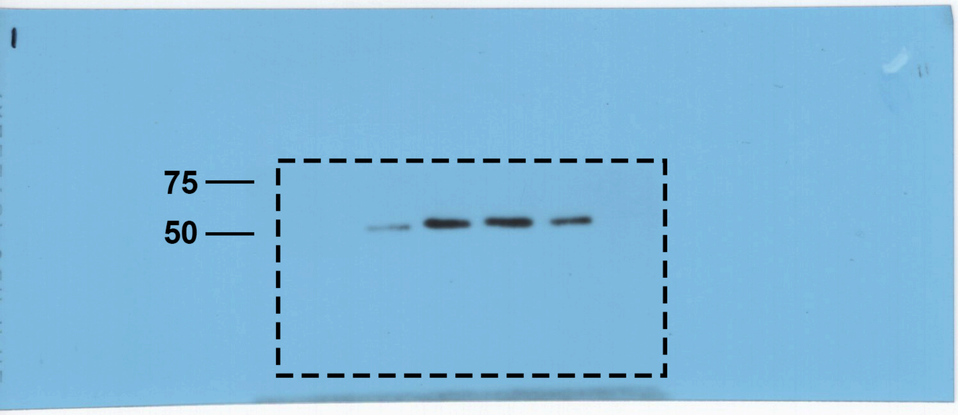

Beclin 1

52 kDa

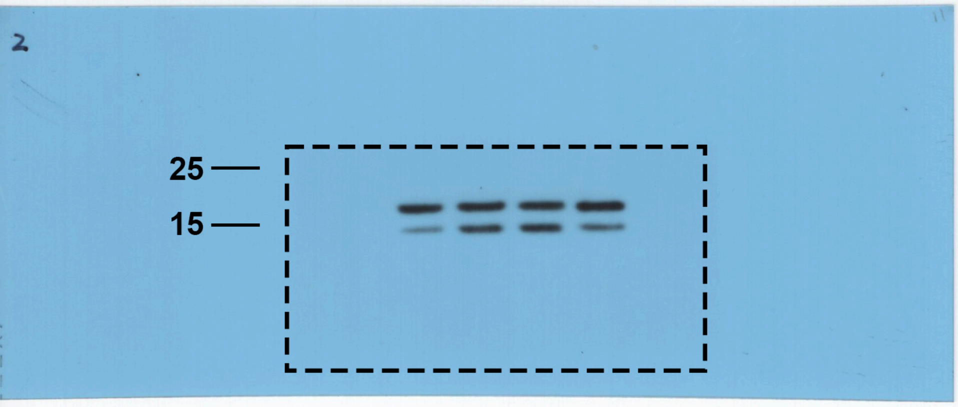

LC3 I

LC3 II

16 kDa

14 kDa

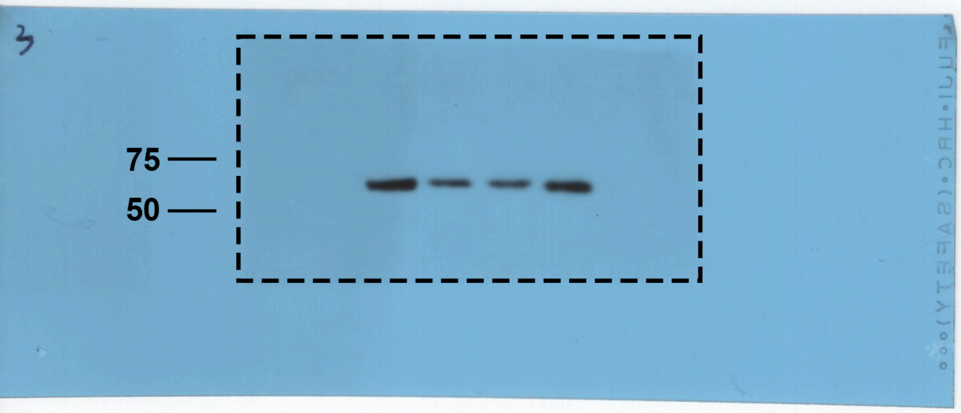

p62

62 kDa

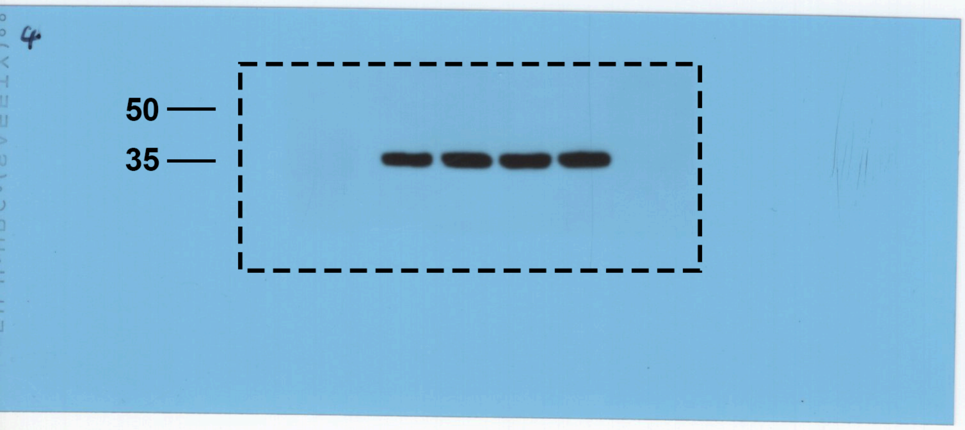

GAPDH

37 kDa

Unedited blots for Figure 2C

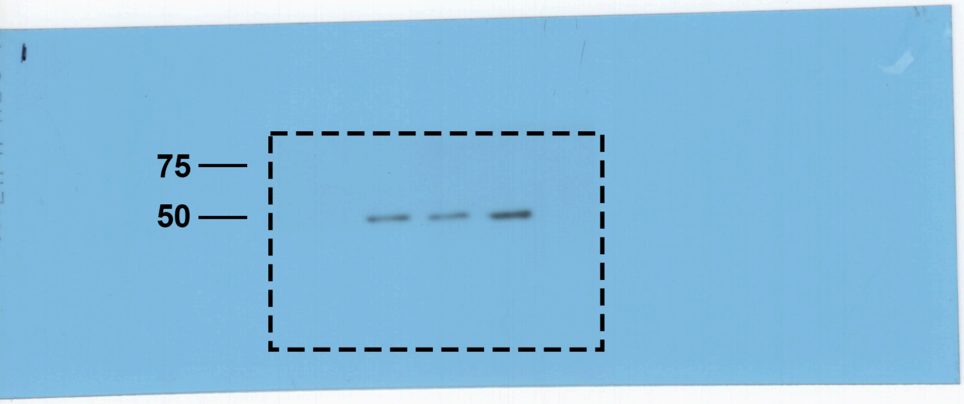

Beclin 1
52 kDa

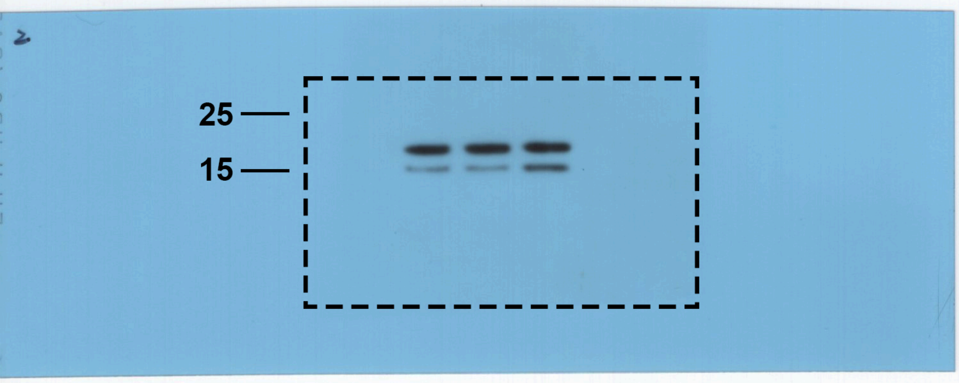

LC3 I
LC3 II
16 kDa
14 kDa

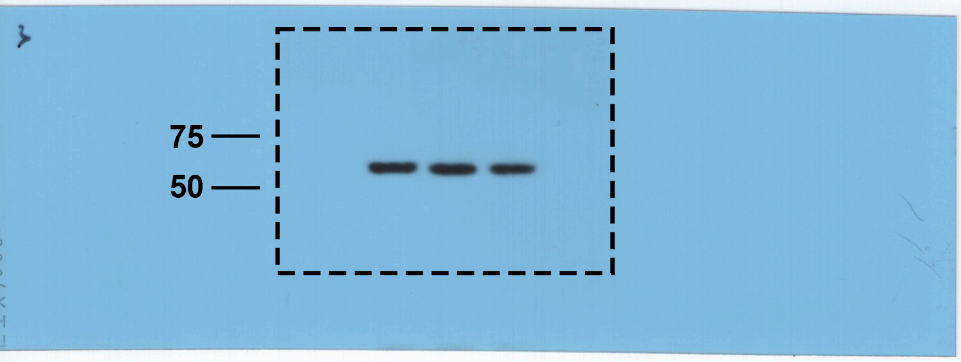

p62
62 kDa

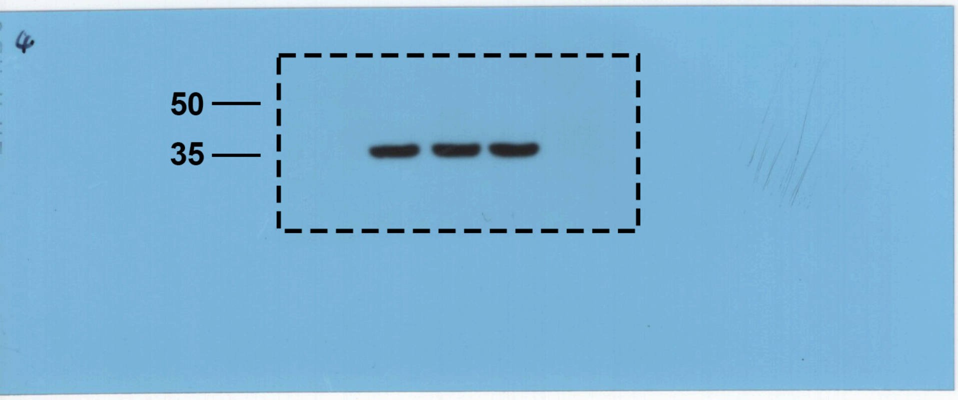

GAPDH
37 kDa

Unedited blots for Figure 3C

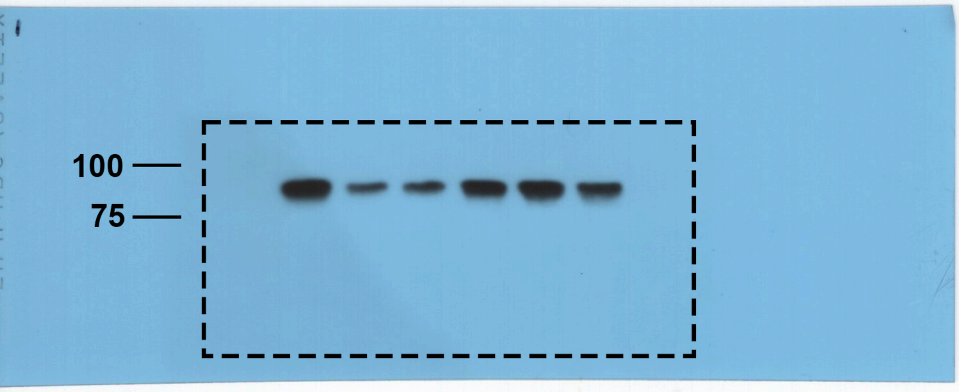

Stat3
88 kDa

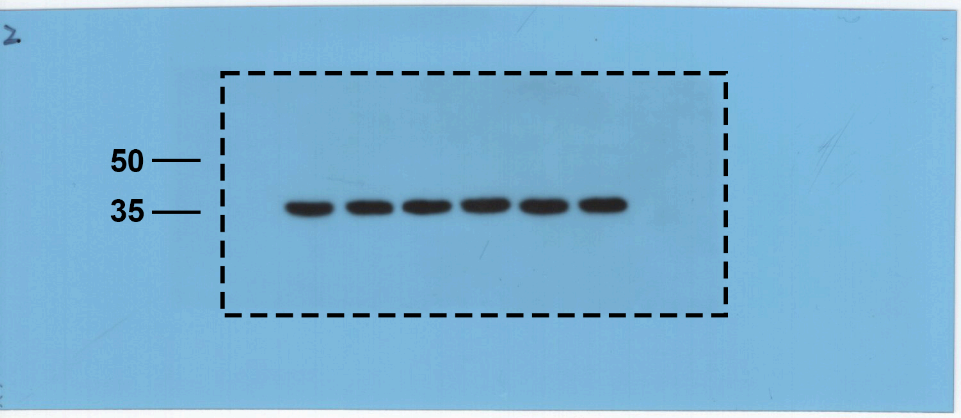

GAPDH
37 kDa

Unedited blots for Figure 4B

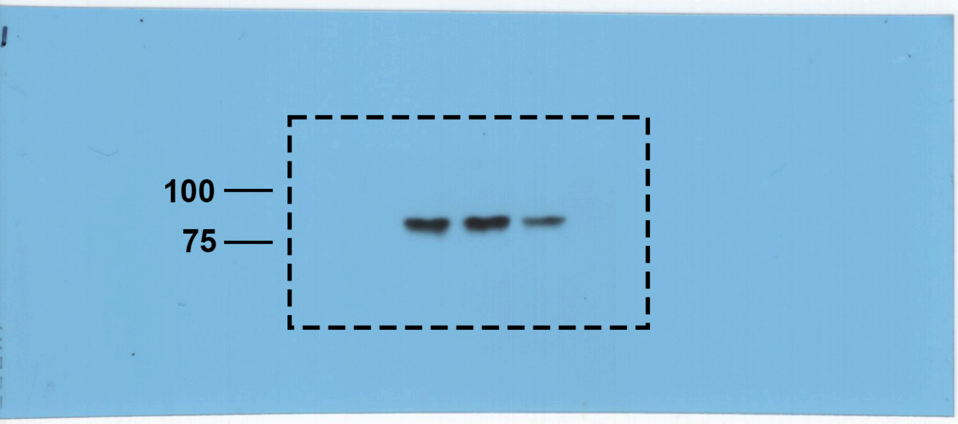

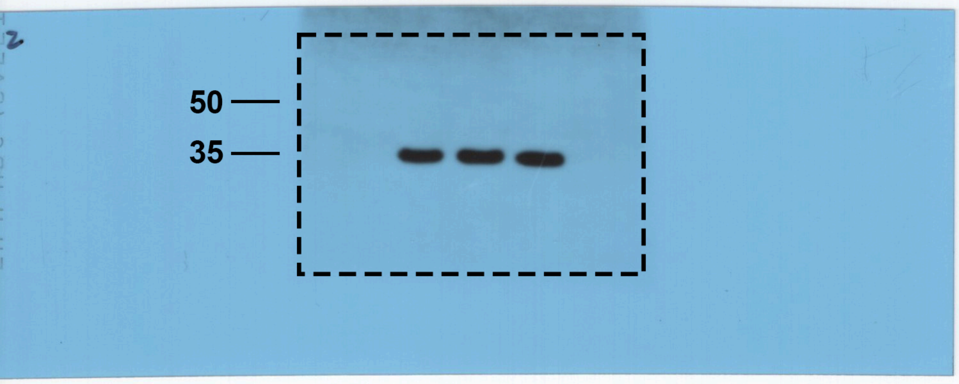

Unedited blots for Figure 4D

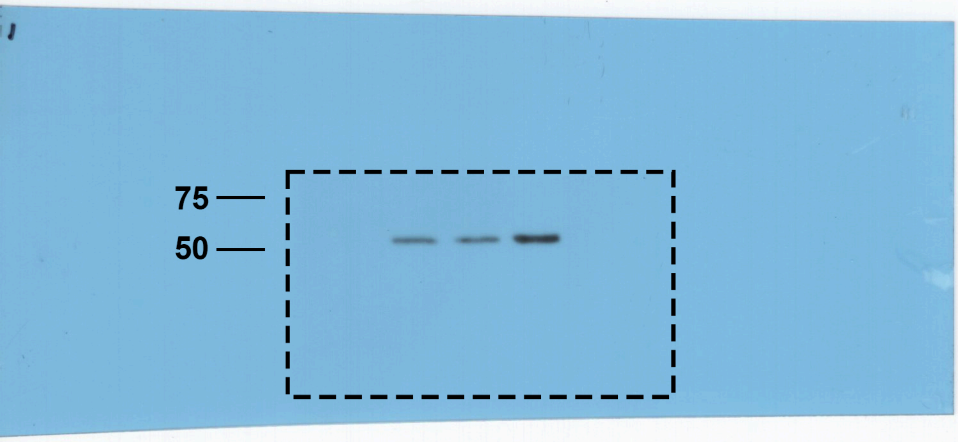

Beclin 1

52 kDa

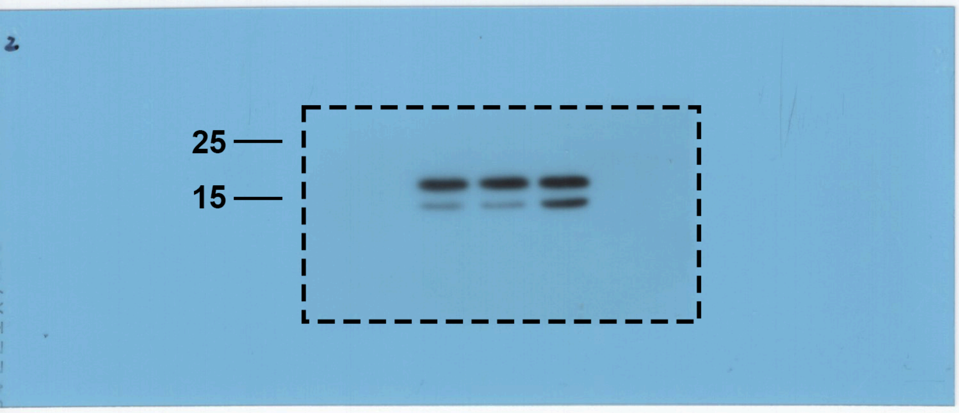

LC3 I

LC3 II

16 kDa

14 kDa

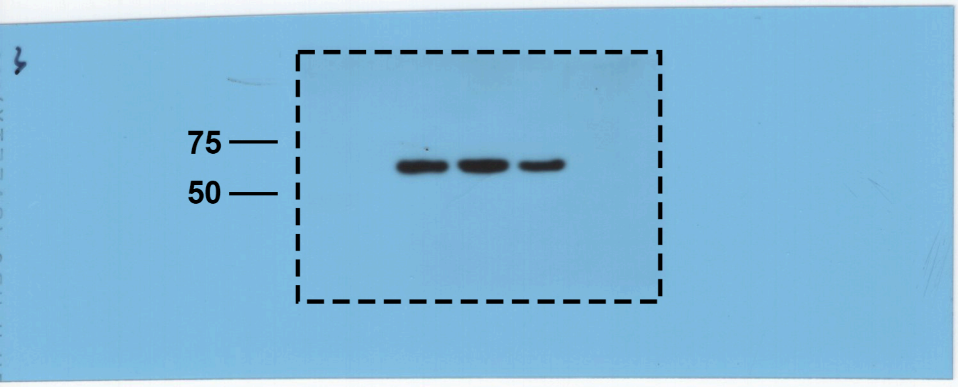

p62

62 kDa

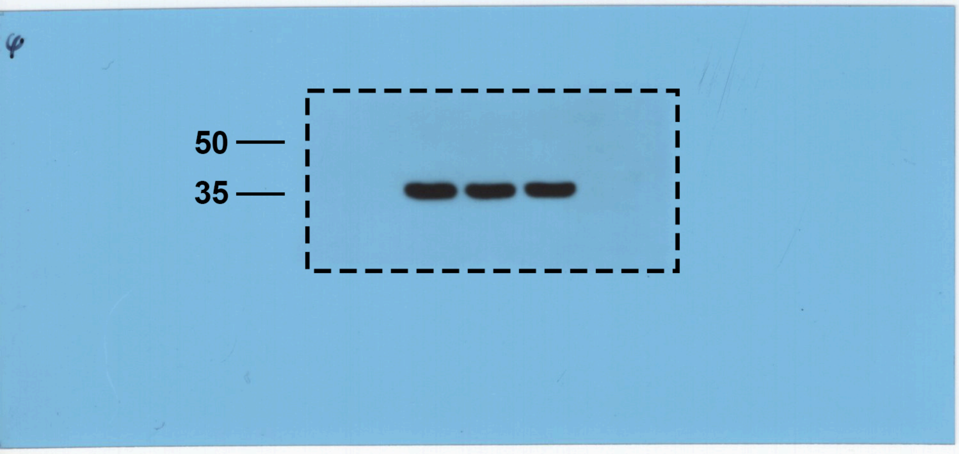

GAPDH

37 kDa

Unedited blots for Figure 5

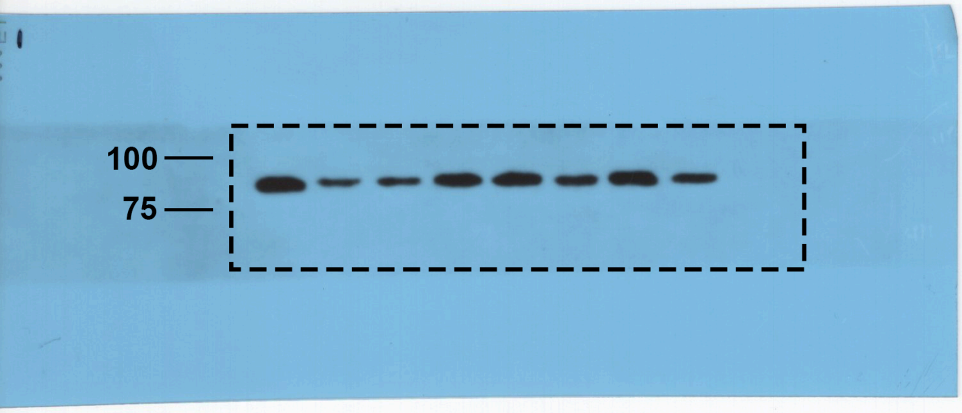

p-PI3K
84 kDa

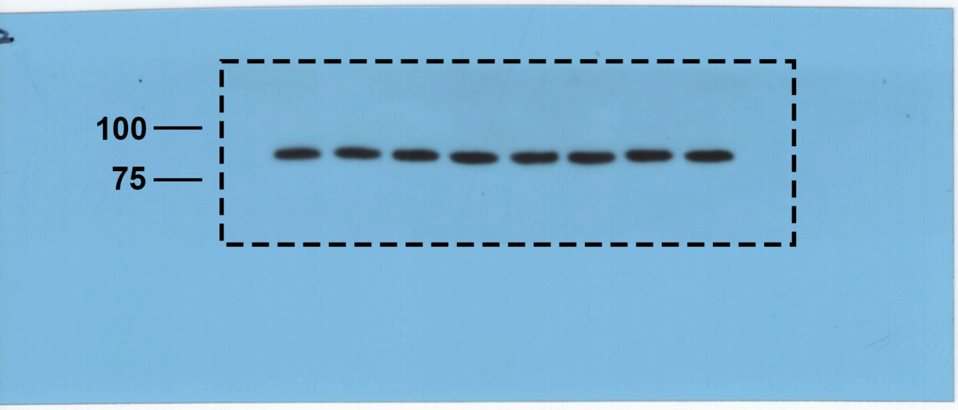

PI3K
84 kDa

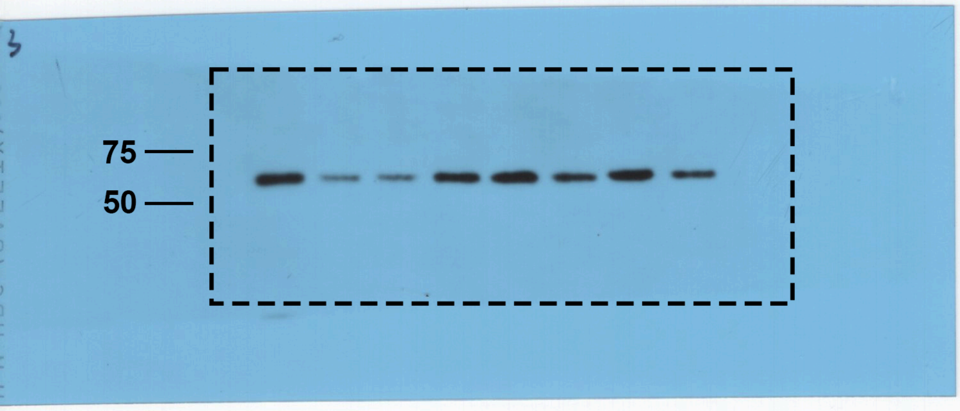

P-AKT
56 kDa

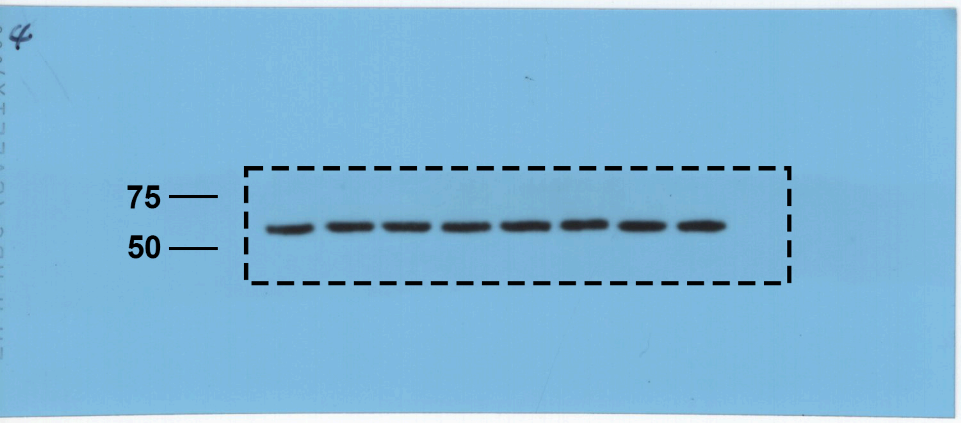

AKT
56 kDa

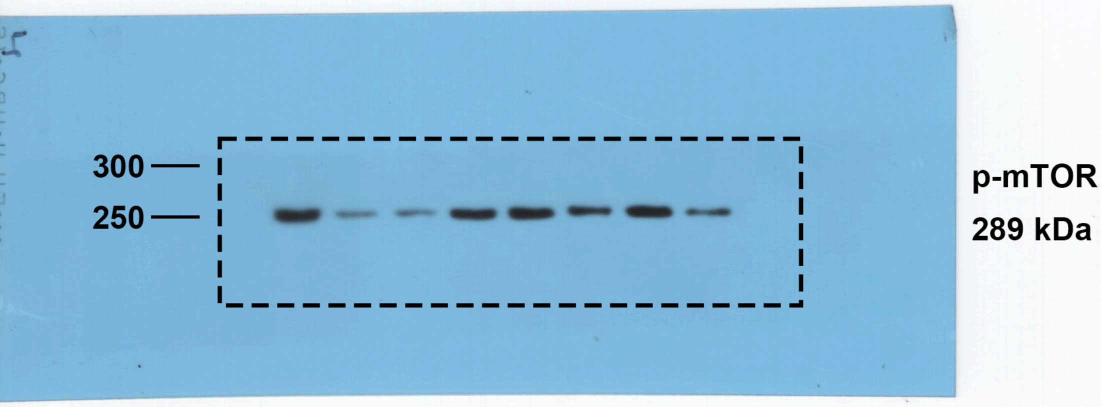
300
250
p-mTOR
289 kDa

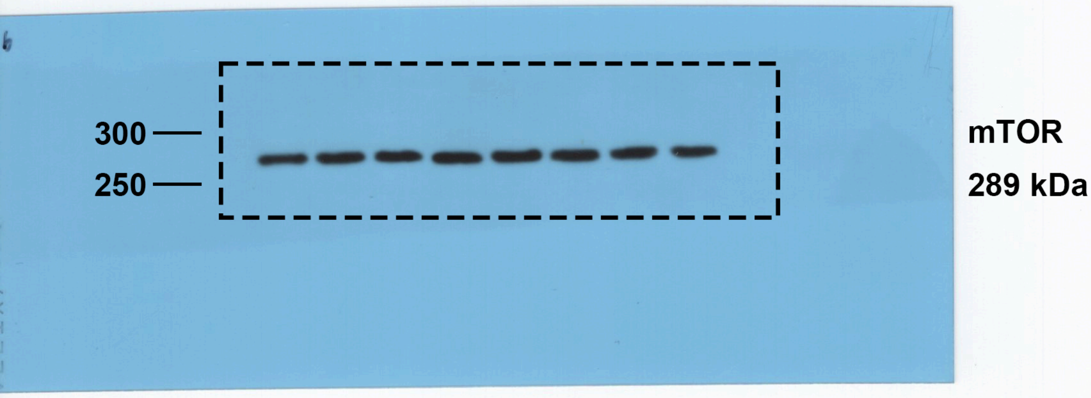
300
250
mTOR
289 kDa

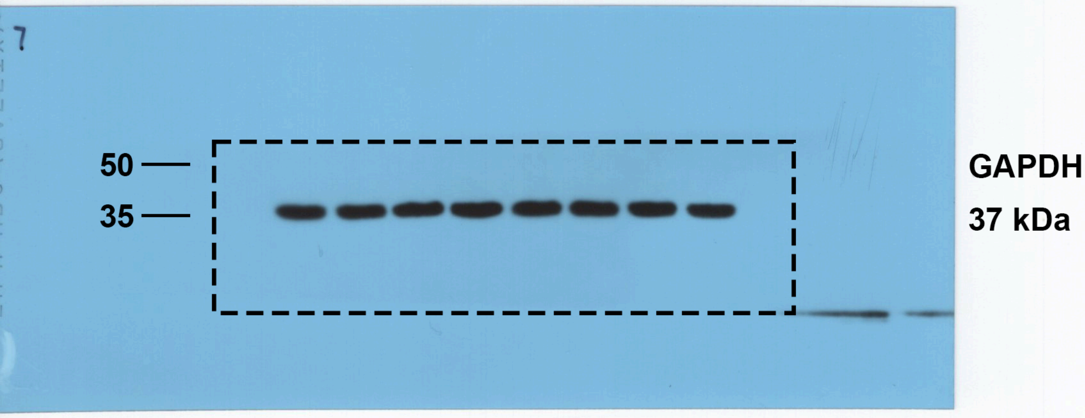
50
35
GAPDH
37 kDa

Unedited blots for Figure 6A

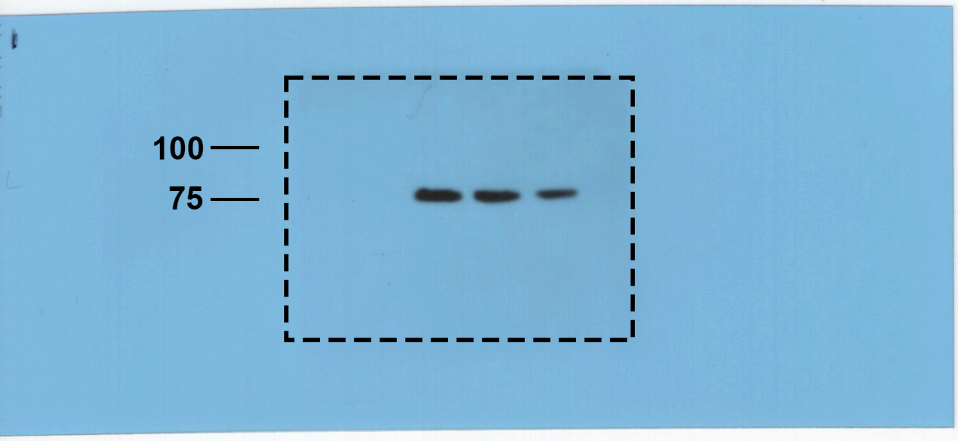

p-PI3K
84 kDa

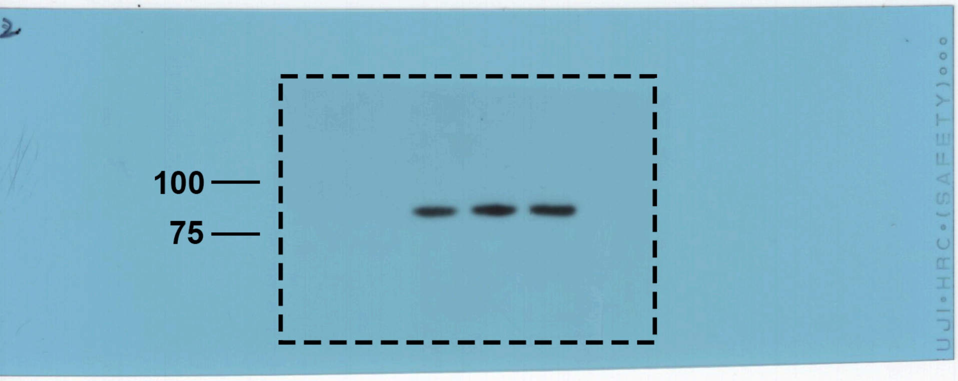

PI3K
84 kDa

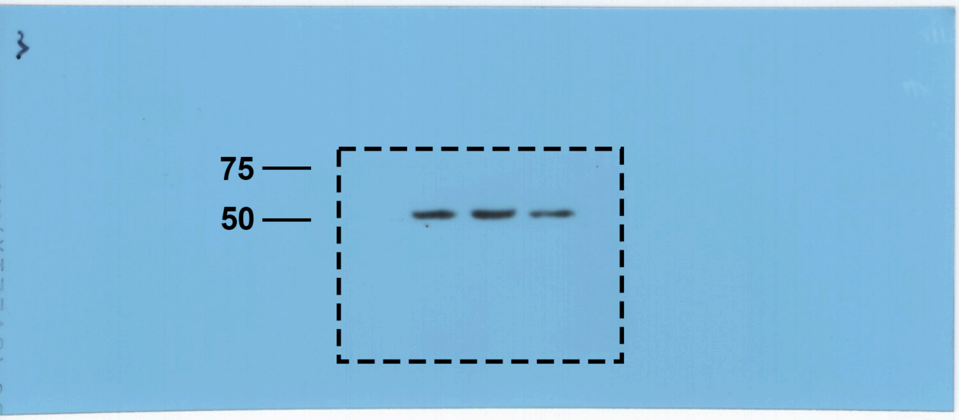

P-AKT
56 kDa

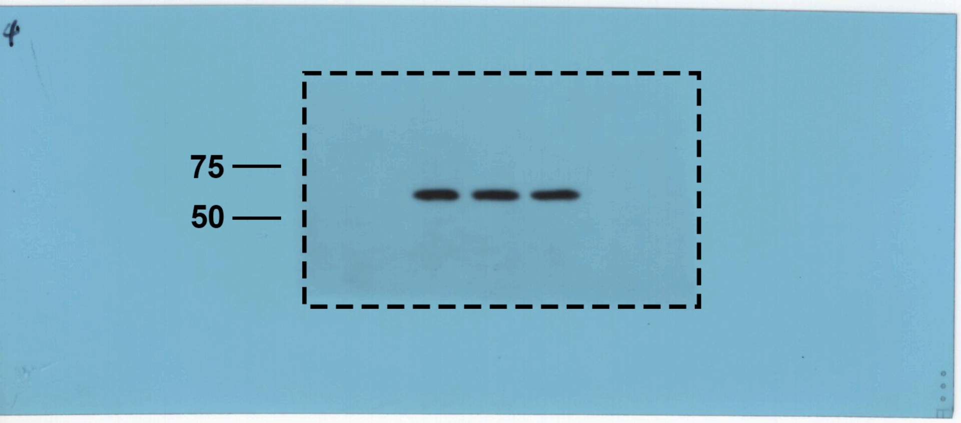

AKT
56 kDa

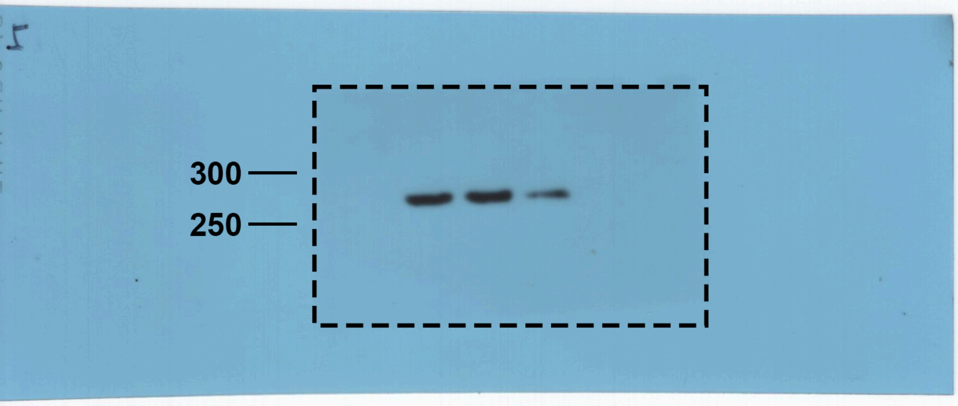

p-mTOR
289 kDa

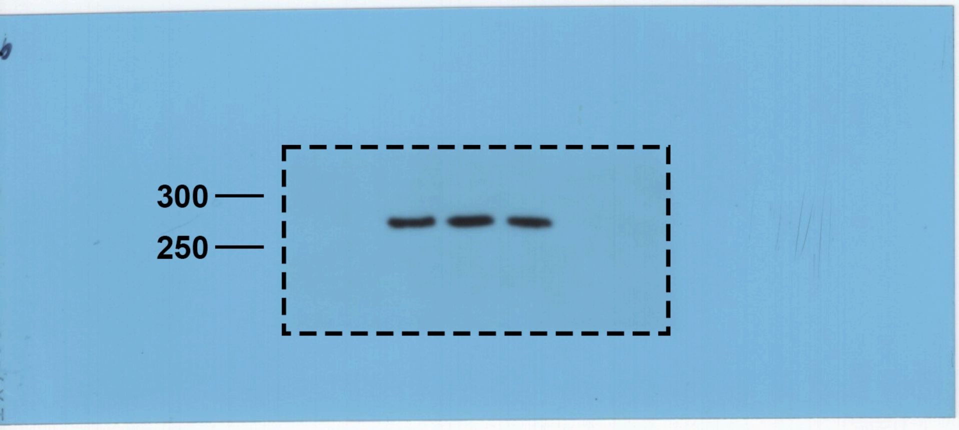

mTOR
289 kDa

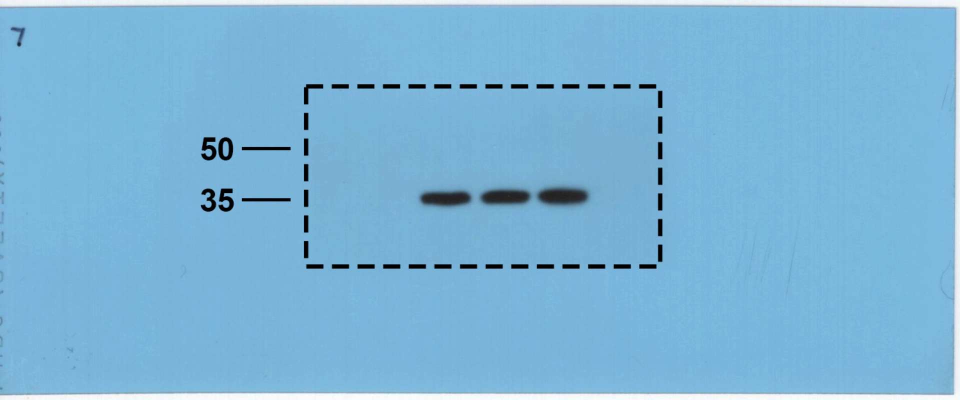

GAPDH
37 kDa

Unedited blots for Figure 6C

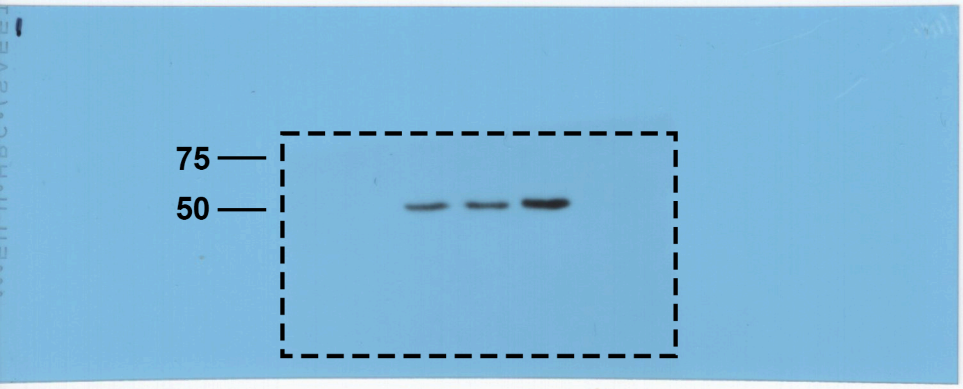

Beclin 1
52 kDa

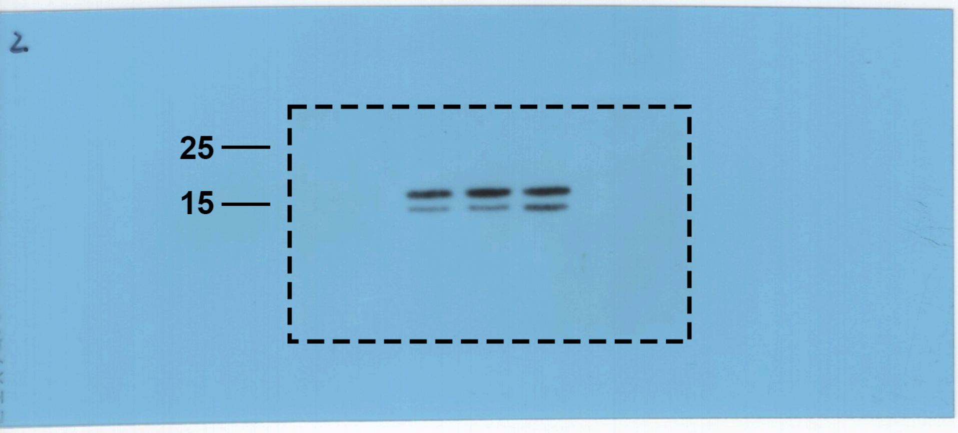

LC3 I
LC3 II
16 kDa
14 kDa

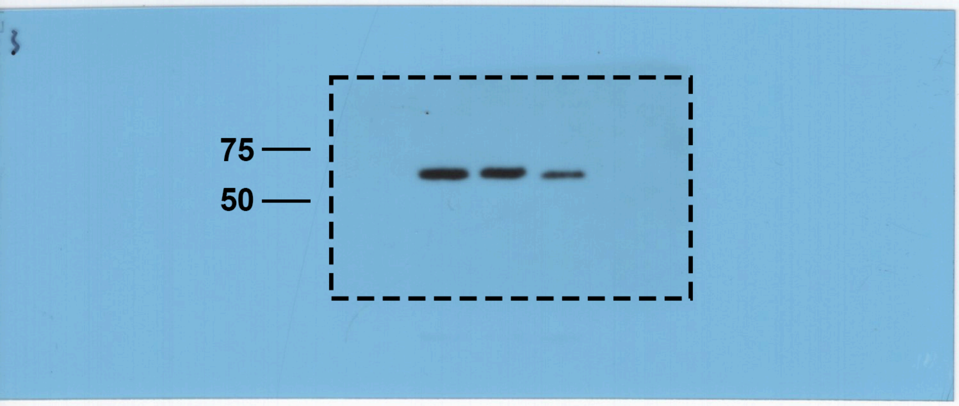

p62
62 kDa

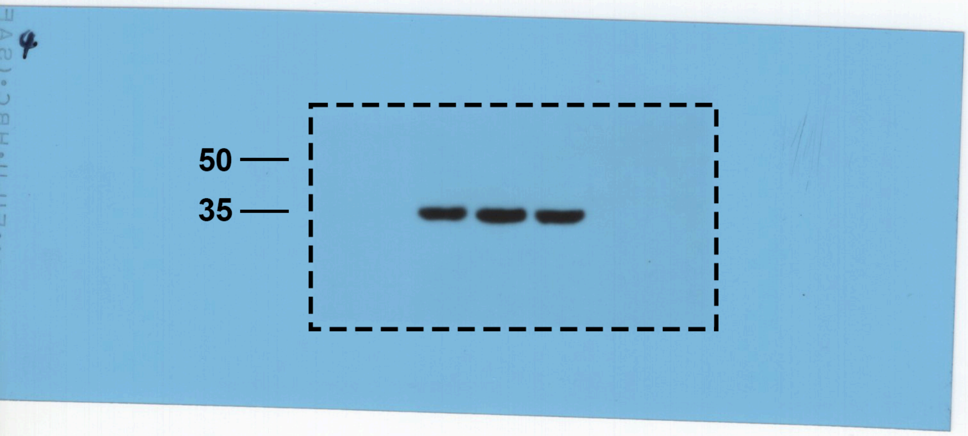

GAPDH
37 kDa
